# The Elastic Fluctuation Tensor: Quantifying Stochastic Fluctuations of the Apparent Stiffness of Non-representative Microstructure Volume Elements

**DOI:** 10.1007/s10659-026-10209-0

**Published:** 2026-06-03

**Authors:** Maximilian Krause, Matti Schneider

**Affiliations:** 1 Institute of Engineering Mathematics, University of Duisburg-Essen, Essen, Germany; 2 Fraunhofer Institute for Industrial Mathematics ITWM, Kaiserslautern, Germany; 3 CENIDE Center for Nanointegration, University of Duisburg-Essen, Essen, Germany; 4 University of Bremen, Bremen, Germany

**Keywords:** Random microstructure, Stochastic homogenization, Symmetry groups, Stiffness fluctuation, Linear elasticity, 74Q05, 74S25, 74B05, 74A60, 74Q20

## Abstract

We introduce the elastic fluctuation tensor to quantify the stochastic fluctuation of the apparent stiffness of finite microstructural volume elements. Typically, in computational homogenization using volume elements of finite size, the apparent stiffness converges to the effective stiffness as the volume element size tends to infinity, such that the material can be approximated as homogeneous on the macroscale. For volume elements of finite size, the apparent stiffness fluctuates on the macroscale. In thermal conductivity homogenization, the fluctuations can be quantified using the fourth-order fluctuation tensor, which computes as the infinite-volume limit of the apparent conductivity covariance, rescaled with the volume. The fluctuation tensor for linear elasticity is of tensor order eight. We show that this fluctuation tensor inherits the symmetry of its ensemble. For instance, rotational statistical symmetry of the ensemble leads to isotropy of the elastic fluctuation tensor. Using results from group representation theory, we define efficient representations of the eighth-order fluctuation tensor for various microstructure symmetry classes and discuss the physical meaning of individual components for the statistically isotropic case. We furthermore leverage symmetry to mitigate numerical errors, thereby reducing the expense of computing the fluctuation tensor. As an example material, we consider polypropylene reinforced by fibers and spherical inclusions. Additionally, we examine polycrystalline copper microstructures. By numerically computing the elastic fluctuation tensor, we confirm theoretical asymptotic convergence rates and symmetry properties. For many of the considered statistically isotropic microstructures, the fluctuations of isotropic stiffness components, which are often the only fluctuations reported, are negligible compared to isotropic fluctuations of the anisotropic stiffness components. Therefore, the full fluctuation tensor must be considered when quantifying the uncertainty of stochastic homogenization.

## Introduction

### State of the Art

Computational homogenization of mechanical properties commonly relies on the use of volume elements (VEs) of finite size to represent the properties of a stochastic ensemble of infinite random microstructures [1, 2]. In engineering practice, the stochastic ensemble is rarely known a priori, and VEs are obtained from direct experimental measurement [3, 4], physical simulation of microstructure formation [5, 6], or are synthetically generated based on geometrical descriptors [7–9]. Computational homogenization proceeds by numerically solving a boundary-value problem for each VE, which is commonly achieved by using the finite element method (FEM) [10] or spectral solvers based on the fast Fourier transform (FFT) [11, 12].

Homogenization of a single VE of finite volume yields the *apparent* properties of the VE, which converge to the *effective* properties of the ensemble in the infinite-volume limit [13–15]. Following Drugan and Willis [16], a VE which is large enough that its apparent properties closely approximate the effective properties is considered a representative volume element (RVE). In computational homogenization, choosing a sufficiently large VE is a trade-off between accuracy and computational expense, as insufficient VE sizes induce both systematic and random errors [17, 18]. Therefore, low errors generally reduce the computational expense associated both with synthetic microstructure generation and solving the boundary-value problem. In recent years, the random fluctuations of the apparent properties are increasingly treated not only as an error to be mitigated, but as quantities of interest in their own right [19, 20].

Kanit et al. [21] used integral ranges [22] to quantify the random error and estimate the RVE size. Since then, computational studies [23, 24] were conducted on integral ranges of physical properties. Jeulin et al. [25] give a detailed overview and argue in favor of using integral ranges to quantify apparent property fluctuations. Integral ranges may be estimated empirically based on variances of the apparent properties computed at different volume sizes [22].

In the statistical volume element (SVE) method, apparent property fluctuations are quantified by computing the apparent properties as random fields. The random fields are retrieved by computing apparent properties of small VEs taken from a larger VE [26]. Since apparent properties computed on finite VEs depend on the imposed boundary condition, applying Dirichlet and Neumann conditions on the VEs leads to two different random fields [27]. The expectations of the random stiffness fields yield the Voigt-Reuss bounds if the SVE is chosen sufficiently small [28]. The SVE method considers fluctuations as SVE-size-dependent properties, not effective properties.

In the quantitative theory of homogenization [29], statistical fluctuations of apparent properties are understood as effective properties of the ensemble. For specific ensembles, Duerinckx et al. [30] proved that, as the VE volume increases, the statistical fluctuations tend to Gaussian random fields with a covariance that scales inversely with the volume [31]. Consequently, Duerinckx et al. [30] quantify the statistical fluctuations by the covariance of apparent properties of finitely-sized VEs multiplied by the VE volume: the apparent fluctuation tensor. Like the apparent properties, the apparent fluctuation tensor converges to an effective fluctuation tensor as the VE size increases. In computational practice, the fluctuation tensor is computed using a large number of synthetic VEs of sufficient size [32]. Nguyen et al. [33] showed that the fluctuation tensor for thermal conductivity problems inherits the statistical symmetry of the microstructure ensemble. By enforcing this symmetry, the representation of the fluctuation tensor is simplified and computational errors are mitigated. The fluctuation tensor approach has been used to quantify fluctuations in conductivity problems, but not yet for linear elasticity.

As the apparent stiffness in linear elasticity is a fourth-order stiffness tensor, the associated fluctuation tensor is of order eight. For fourth-order tensors such as the elastic stiffness, the algebraic consequences of rotational symmetry are well-explored [34]. Consequently, fourth-order tensors allow for efficient representations [35] and intuitive visualization [36]. The literature on eighth-order tensors is more sparse. While tables of crystallographically symmetric eighth-order tensors were published [37], these do not take into account the index symmetries present in the fluctuation tensor. Tensorial formulations of group representation theory [38, 39] allow for the definition of rotationally symmetric bases to simplify tensor representations for tensors of arbitrary order [40].

### Contributions

For homogenization problems in thermal conductivity, the covariance of the apparent property scaled by the volume was shown to converge to the fourth-order fluctuation tensor in the infinite volume limit, though existing results are limited to specific ensembles [30]. To similarly quantify apparent stiffness fluctuations in linear elasticity, we study the natural equivalent in this setting: the eighth-order stiffness fluctuation tensor. We wish to emphasize that we follow a computationally driven approach here - we do not have a mathematical proof justifying the existence of the fluctuation tensor in elasticity. Rather, we hypothesize that the properties valid for the conductivity setting transfer to linear elasticity and report on dedicated computational investigations.

Continuum mechanics often deals with fourth-order minor-symmetric tensors, which are commonly represented by ${6 \times 6}$ matrices. Since a symmetric ${6 \times 6}$ matrix like the stiffness has 21 independent components, the covariance of the stiffness can in principle be represented by a symmetric ${21 \times 21}$ matrix. However, such a representation must be carefully chosen so that its components have immediate physical meaning and symmetries can be recognized at a glance. We develop a matrix representation of the eighth-order fluctuation tensor by harnessing the harmonic basis formalism.

Statistical symmetries of the microstructure ensemble imply corresponding symmetries of the apparent properties. This symmetry allows for considerable simplification of effective stiffness representations in practice, and can also be utilized to reduce the errors inherent in computational homogenization. We show that microstructural symmetry implies fluctuation tensor symmetry. For the special case of statistical isotropy, we demonstrate that the number of independent components of the fluctuation tensor reduces to seven. Using symmetry, we develop an efficient strategy for computing the fluctuation tensor.

During the revision of the manuscript we became aware of related work [41] which exploits an eighth-order tensorial stiffness covariance, as well. However, Shivanand et al. [41] use the stiffness covariance for modeling random stiffness fields where the covariance serves as an input which quantifies anisotropic fluctuations. In contrast, the fluctuation tensors we investigate and compute are outputs of traditional stochastic homogenization analyses. In particular, both approaches are naturally compatible and synergistic: The fluctuations tensors we produce serve as natural parameters for the stochastic setting.

For the examples of polypropylene with E-glass fillers and polycrystalline copper, we compute the associated linear elastic fluctuation tensors. To showcase the effect of microstructural morphology, we discuss spherical and cylindrical E-glass inclusions. We assume statistical isotropy, and discuss that using cubic volume elements breaks the symmetry of the statistical ensemble. Using symmetry conditions on the eighth-order fluctuation tensor, we separate the error into symmetric and anisotropic parts, which provides effective error mitigation strategies.

## Homogenization of Linear Elasticity

### Stochastic Homogenization

An infinitely large microstructure is described by a stiffness field 2.1 which specifies a minor- and major-symmetric fourth-order tensor  for every continuum point . For convenience, we assume that the stiffness field satisfies the pointwise two-sided bounds 2.2 with positive real numbers $a$ and $b$. Additionally, we assume that the field ℂ is a measurable function ${\mathbb{C}} \in L^{ \infty}$ and denote the set of all stiffness fields ℂ as 2.3

In stochastic homogenization, the field ℂ is a random quantity. On the set $\mathcal{C}$ with the event space ℱ, an ensemble is given by a probability measure, which we consider as a function associating a real number to every measurable set of events 2.4 For the measure $\mu $ to be a probability measure, it must be non-negative, normalized and countably additive [42]. Using the measure $\mu $, we define the corresponding ensemble average

2.5 $$ \langle F\rangle = \int _{\mathcal{C}} F( \mathbb{C} ) {\,\mathrm{d}}{\mu ( \mathbb{C} )} $$

for all $\mu $-integrable functions . In the following, we are interested in the effective elastic properties of ensembles which are stationary and ergodic. An ensemble of stiffness fields is stationary if its statistical properties do not change under spatial shifts, i.e., the ensemble is statistically homogeneous. In engineering practice, ergodicity encodes that sufficiently large volumes have the same statistical properties as the ensemble. This notion can be made mathematically rigorous using group theory [14].

Given an applied macroscopic strain , each random stiffness field ℂ yields a unique corrector field 2.6 of sublinear growth as the solution of the corrector form of the linear balance of momentum 2.7 The associated random strain and stress fields

2.8 $$\begin{aligned} \boldsymbol{\varepsilon } _{ \bar{ \boldsymbol{\varepsilon } }} &= \bar{ \boldsymbol{\varepsilon } } + \nabla ^{s} \boldsymbol{u} _{ \bar{ \boldsymbol{\varepsilon } }} \end{aligned}$$

2.9 $$\begin{aligned} \boldsymbol{\sigma } _{ \bar{ \boldsymbol{\varepsilon } }} &= \mathbb{C} [ \bar{ \boldsymbol{\varepsilon } } + \nabla ^{s} \boldsymbol{u} _{ \bar{ \boldsymbol{\varepsilon } }}] \end{aligned}$$

are stationary [43, 44]. The homogeneous effective stress of the ensemble computes as

2.10 $$ \bar{ \boldsymbol{\sigma } }_{ \bar{ \boldsymbol{\varepsilon } }} = \langle \mathbb{C} [ \bar{ \boldsymbol{\varepsilon } } + \nabla ^{s} \boldsymbol{u} _{ \bar{ \boldsymbol{\varepsilon } }}] \rangle . $$

By the linearity of the differential equation (2.7), we may use the effective stress and strain to define the effective stiffness  implicitly via the rule 2.11 Classically, the goal of linear elastic homogenization for random composites is computing the effective stiffness [45].

### Computational Homogenization of Periodized Ensembles

To avoid infinitely large domains, stochastic homogenization typically proceeds by restricting to a cubic domain 2.12 and considering stiffness fields  which are periodic on the domain $Y_{L}$. The $Y_{L}$-periodic stiffness fields $\mathbb{C} _{ L }$ obeying the bounds (2.2) form the subset

2.13 $$ \mathcal{C}_{ L }\subseteq \mathcal{C} $$

of the set of stiffness fields $\mathcal{C}$ (2.3).

In the periodic setting, it is sufficient to solve the balance of linear momentum (2.7) on the domain $Y_{L}$ (2.12)

2.14 $$ {\mathrm{div }}\left ( \mathbb{C} _{ L }( {\boldsymbol{x}} ) [ \bar{ \boldsymbol{\varepsilon } } + \nabla ^{s} \boldsymbol{u} _{ L , \bar{ \boldsymbol{\varepsilon } }}( {\boldsymbol{x}} )] \right ) = \boldsymbol{0} , \quad {\boldsymbol{x}} \in Y_{L}, $$

with periodic boundary conditions

2.15 $$\begin{aligned} \boldsymbol{u} ( {\boldsymbol{x}} _{+}) &= \boldsymbol{u} ( {\boldsymbol{x}} _{-}), \end{aligned}$$

2.16 $$\begin{aligned} \boldsymbol{\sigma } ( {\boldsymbol{x}} _{+}) \boldsymbol{n} ( {\boldsymbol{x}} _{+}) &= - \boldsymbol{\sigma } ( {\boldsymbol{x}} _{-}) \boldsymbol{n} ( {\boldsymbol{x}} _{-}), \end{aligned}$$

for opposing points ${\boldsymbol{x}} _{+}$, ${\boldsymbol{x}} _{-}$ on the boundary $\partial Y_{L}$, and where $\boldsymbol{n} $ denotes the outward-pointing normal vector field. We denote the unique solution of the balance of linear momentum (2.14) by $\boldsymbol{u} _{ L , \bar{ \boldsymbol{\varepsilon } }}$. As with the effective stress (2.10) of the stochastic ensemble, we define a homogeneous apparent stress

2.17 $$ \boldsymbol{\sigma } ^{ \mathrm{app} }_{ L , \bar{ \boldsymbol{\varepsilon } }} = \frac{1}{L^{3}} \int _{Y_{{{ L }}}} \mathbb{C} _{ L }( {\boldsymbol{x}} )[ \bar{ \boldsymbol{\varepsilon } } + \nabla ^{s} \boldsymbol{u} _{ L , \bar{ \boldsymbol{\varepsilon } }}( {\boldsymbol{x}} )] {\,\mathrm{d}}V( {\boldsymbol{x}} ). $$

Since the balance of linear momentum (2.14) is a linear differential equation, the relationship between apparent stresses (2.17) and the applied effective strain $\bar{ \boldsymbol{\varepsilon } }$ is linear. This linear relation is quantified by the apparent stiffness tensor $\mathbb{C} ^{\mathrm{app}} _{ L }$ of a single periodic field $\mathbb{C} _{ L }$, defined via the implicit relation 2.18 Periodic homogenization theory [46] applied to a *single* realization shows that the apparent stiffness $\mathbb{C} ^{\mathrm{app}} _{ L }$ is positive-definite as well as minor and major symmetric .

The apparent stiffness $\mathbb{C} ^{\mathrm{app}} _{ L }$ quantifies the stiffness of a particular sample and comes with two sources of error. For a start, this stiffness depends on the particular sample, reflecting the randomness of the sampling process. Moreover, there is another source of error which is more subtle: The apparent stiffness also depends on the size of the sample. Indeed, the sample size might be too small to capture long-range features of the ensemble. In contrast, the effective stiffness $\bar {\mathbb{C}} $ describes the mechanical response of the entire ensemble of the random material. It can be thought of corresponding to the stiffness of an infinitely large microstructure. The effective stiffness captures all long range correlations and also all fluctuations. In particular, it is a deterministic quantity.

To connect these two tensors, we consider a periodic ensemble on the set of periodic stiffnesses $\mathcal{C}_{ L }$ (2.13), with an event space $\mathcal{F}_{ L }$ and a probability measure $\mu _{ L }$. Naturally, the periodic ensemble defines an ensemble average

2.19 $$ \langle F\rangle _{L} = \int _{\mathcal{C}_{{{ L }}}} F( \mathbb{C} _{{{ L }}}) { \,\mathrm{d}}{\mu _{L}( \mathbb{C} _{{ { L }}})} $$

for any $\mu _{ L }$-integrable function $F$.

When computing on a finite volume $\mu _{ L }$, we approximate a non-periodic ensemble with measure $\mu $ by the periodized measure 2.20 i.e., by conditioning the measure on periodicity. A precise definition of this periodization strategy is given by Gloria et al. [47, Remark 5]. Periodization is particularly straightforward for ensembles where periodic realizations can be constructed directly without the need for rejection sampling [15]. For instance, periodic construction algorithms are available for the polycrystals [48] and non-overlapping inclusion ensembles considered in this work [49, 50].

The periodic measure $\mu _{L}$ vanishes on all sets containing only non-periodic stiffness fields, is normalized, and inherits stationarity from the measure $\mu $ [15]. The apparent stiffness of the periodized approximation converges to the effective stiffness in the infinite-size limit [51]

2.21 $$ \mathbb{C} ^{\mathrm{app}} _{L} \longrightarrow \bar {\mathbb{C}} \quad \mathrm{as} \quad L \longrightarrow \infty \quad \text{almost surely}. $$

Since the periodized ensemble is generally not ergodic, the apparent stiffness $\mathbb{C} ^{\mathrm{app}} _{L}$ is a random quantity, as opposed to the mean apparent stiffness $\langle \mathbb{C} ^{\mathrm{app}} _{L} \rangle $. As observed by Gloria et al. [52], the square of the error induced by periodization

2.22 $$ e^{2} = \langle \| \mathbb{C} ^{ \mathrm{app}} _{L} - \bar {\mathbb{C}} \|^{2}\rangle $$

decomposes into the square of the systematic and the random error

2.23 $$\begin{aligned} e_{\mathrm{sys}}^{2} &= \| \bar {\mathbb{C}} - \langle \mathbb{C} ^{\mathrm{app}} _{L} \rangle \|^{2}, \end{aligned}$$

2.24 $$\begin{aligned} e_{\mathrm{rand}}^{2} &= \langle \| \mathbb{C} ^{\mathrm{app}} _{L} - \langle \mathbb{C} ^{\mathrm{app}} _{L} \rangle \|^{2}\rangle . \end{aligned}$$

The square of the random error $e_{\mathrm{rand}}^{2}$ (2.24) arises as the trace of the eighth-order covariance tensor

2.25 $$ \operatorname{cov}( \mathbb{C} ^{ \mathrm{app}} _{ L }) = \left\langle \left ( \mathbb{C} ^{\mathrm{app}} _{ L }- \langle \mathbb{C} ^{\mathrm{app}} _{ L }\rangle _{L} \right ) \otimes \left ( \mathbb{C} ^{\mathrm{app}} _{ L }- \langle \mathbb{C} ^{\mathrm{app}} _{ L }\rangle _{L} \right )\right\rangle _{L} , $$

which converges to zero at an asymptotic rate of $\mathcal{O}(L^{-3})$ in the three-dimensional case for sufficiently well-behaved ensembles [47]. Following the work of Duerinckx et al. [30] on thermal conductivity, we define the apparent fluctuation tensor

2.26 $$ {\mathbb{Q}}^{8} _{ L }= L^{3} \left\langle \left ( \mathbb{C} ^{ \mathrm{app}} _{ L }- \langle \mathbb{C} ^{\mathrm{app}} _{ L } \rangle _{L} \right ) \otimes \left ( \mathbb{C} ^{\mathrm{app}} _{ L }- \langle \mathbb{C} ^{\mathrm{app}} _{ L }\rangle _{L} \right )\right\rangle _{L} . $$

By analogy to the work of Duerinckx et al. [30] on thermal conductivity, we expect the apparent fluctuation tensor (2.26) to converge asymptotically towards the effective fluctuation tensor

2.27 $$ {\mathbb{Q}}^{8} _{ L } \longrightarrow {\mathbb{Q}}^{8} \quad \mathrm{as} \quad L \longrightarrow \infty . $$

For the thermal conductivity fluctuation tensor, the convergence relation (2.27) was proved for specific ensembles [30], with the convergence rate

2.28 $$ \| {\mathbb{Q}}^{8} _{ L }- {\mathbb{Q}}^{8} \| \propto \mathcal{O}(L^{-\frac{3}{2}}) $$

up to logarithmic terms. For elastic problems, we are not aware of an explicit proof of the relation (2.27). However, we do not expect fundamental difficulties in extending the work of Duerinckx et al. [30] to this case. As the analytical framework does not cover the industrial ensembles of interest to us, we resort to computational studies and verify the convergence rate (2.28) empirically.

Similarly to the effective stiffness, the elastic fluctuation tensor does not depend on the boundary conditions or the length of the RVE, and therefore quantifies properties of the ensemble at large, not a particular periodic realization. It therefore allows to robustly quantify the uncertainty of imperfect scale transitions, i.e., the effect of microstructure characteristic lengths being close to the geometry of the part scale. In the following, we discuss properties of the elastic fluctuation tensor ${\mathbb{Q}}^{8} $ and devise computational strategies for evaluating example microstructures.

### Statistical Symmetry

To simplify and validate numerically obtained results, we discuss how microstructural symmetry influences the effective and apparent properties. Generally, we are interested in rotational symmetry, which is governed by the action of a rotation

2.29 $$ \boldsymbol{R} \in { \mathit{SO}(3)} $$

on the quantities under consideration. We choose the bold symbol $\boldsymbol{R} $ to emphasize that rotations are classically understood as proper orthogonal tensors of second order. This relationship is seen in the action of the rotation $\boldsymbol{R} $ on a first-order tensor ,

2.30 $$ \boldsymbol{R} \star {\boldsymbol{x}} = \boldsymbol{R} {\boldsymbol{x}} . $$

The action of rotations on other objects follows from a general principle. For a general map

2.31 $$ L: A \rightarrow B, $$

between sets $A$ and $B$ closed under rotation, we define the action of rotations on the map itself as

2.32 $$ ( \boldsymbol{R} \star L)(X) \rightarrow \boldsymbol{R} \star L({ \boldsymbol{R} }^{\mathsf{T}} \star X) \quad \text{for all} \quad X \in A, $$

using ${ \boldsymbol{R} }^{\mathsf{T}}$ for the inverse rotation. The axioms of identity and compatibility are quickly verified in this general setting, showing that the operation ⋆ is indeed a group action.

Tensors may be interpreted as linear mappings between tensor spaces. For example, the tensor space  of order $(n+1)$ may be interpreted as a space of linear mappings 2.33 The action of a rotation $\boldsymbol{R} $ on an $n$-th order tensor ${\mathbb{V}}^{n} $ follows inductively by using Eq. (2.32), leading to the Rayleigh product

2.34 $$ ( \boldsymbol{R} \star {\mathbb{V}}^{n} )_{i_{1} \ldots i_{n}} \hat{=} R_{i_{1} j_{1}} \cdots R_{i_{n} j_{n}} V_{j_{1} \ldots j_{n}}, $$

using index notation with Einstein’s summation convention. The action on a stiffness field ℂ (2.1) computes as

2.35 $$ \left ( \boldsymbol{R} \star \mathbb{C} \right )( {\boldsymbol{x}} ) = \boldsymbol{R} \star \mathbb{C} ({ \boldsymbol{R} }^{\mathsf{T}} {\boldsymbol{x}} ). $$

For a set $M \in \mathcal{F}$ of stiffness fields ℂ, we define the rotation action

2.36 $$ \boldsymbol{R} \star M = \left \{ \boldsymbol{R} \star \mathbb{C} \,\middle |\, \mathbb{C} \in M \right \}. $$

Finally, the rotation action on a probability measure $\mu $ is implicitly defined by

2.37 $$ ( \boldsymbol{R} \star \mu )(M) = \mu ({ \boldsymbol{R} }^{\mathsf{T}} \star M) \quad \text{for all} \quad M \in \mathcal{F}, $$

which requires that the $\sigma $-algebra ℱ is conserved under all rotations $\boldsymbol{R} \in { \mathit{SO}(3)}$.

An object $A$ is called symmetric with respect to a subgroup

2.38 $$ S \subseteq { \mathit{SO}(3)} $$

if the condition

2.39 $$ \boldsymbol{R} \star A = A \quad \text{holds for all rotations} \quad \boldsymbol{R} \in S . $$

Specifically, a microstructure ensemble is *statistically symmetric* if its associated probability measure $\mu $ satisfies Eq. (2.39) with a symmetry group (2.38). The largest such symmetry group is consequently denoted as the ensemble symmetry group $S _{\mu}$. An ensemble which is rotationally invariant or *statistically isotropic* has the symmetry group

2.40 $$ S _{\mu }= \mathit{SO}(3), $$

whereas a fully anisotropic ensemble has the trivial symmetry group

2.41 $$ S _{\mu }= \{ \boldsymbol{1}\}, $$

with the symbol $\boldsymbol{1} $ denoting the neutral element of the rotation group $\mathit{SO}(3)$.

Statistical symmetry of the ensemble implies a corresponding symmetry of the effective stiffness [14, 33, 42]. Formally, the effective stiffness of a statistically symmetric ensemble satisfies the symmetry condition (2.39) with the symmetry group $S _{\bar {\mathbb{C}} } \subseteq { \mathit{SO}(3)}$, which fulfills the condition

2.42 $$ S _{\bar {\mathbb{C}} } \supseteq S _{\mu}. $$

The effective property symmetry group $S _{\bar {\mathbb{C}} }$ is a superset ⊇ instead of being equal = to the ensemble symmetry group $S _{\mu}$ since the effective stiffness symmetry may exceed the ensemble symmetry in general. In stiffness homogenization, a prominent example is Hashin et al.’s [53] assemblage of coated spheres composed of isotropic phases. Hashin’s assemblage always yields an isotropic effective stiffness tensor. This symmetry holds even if the microstructural arrangement of the spheres forms an anisotropic geometry, such as a regular lattice, as the coated spheres impose no heterogeneous fields on the surrounding matrix and therefore do not interact.

A periodized ensemble is constrained in its symmetry both by the underlying random ensemble and the periodic domain [33]. Formally, the periodic symmetry group is given by the intersection

2.43 $$ S _{\mu _{ L }} = S _{\mu }\cap S _{Y_{L}}. $$

Since we consider cubic domains (2.12), the group $S _{Y_{L}}$ is given by the cubic symmetry group. Consequently, even for fully isotropic ensembles with $S _{\mu }= { \mathit{SO}(3)}$, the periodic symmetry group is expected to be cubic. There are exceptions, of course, such as the trivial case of a homogeneous stiffness, where the periodized ensemble is isotropic regardless of domain.

The periodic symmetry group implies the symmetry of the *mean* apparent stiffness [33]

2.44 $$ S _{ \langle \mathbb{C} ^{ \mathrm{app}} \rangle _{L} } \supseteq S _{\mu _{ L }}. $$

The general symmetry observations made above also follow for the effective and apparent fluctuation tensors. The effective fluctuation tensor enjoys the ensemble symmetry

2.45 $$ S _{ {\mathbb{Q}}^{8} } \supseteq S _{ \mu}, $$

while the apparent fluctuation tensor inherits the periodic symmetry

2.46 $$ S _{ {\mathbb{Q}}^{8} _{ L }} \supseteq S _{\mu _{ L }}, $$

only. For the readers’ convenience, the arguments for the validity of the statements (2.45) and (2.46) are collected in Appendix.

## A Symmetry-Aware Notation for the Elastic Fluctuation Tensor

### Harmonic Bases of Tensor Spaces

To encode the fluctuation tensor as a matrix or vector, we require a tensorial basis convention. As we wish to represent rotational symmetries of fluctuation tensors efficiently, we choose the harmonic basis [40]. In this section, we outline the derivation of this basis in the general case by applying representation theory to products of tensor spaces. Subsequently, we work out the basis convention for the space of fluctuation tensors using an incremental construction via bases for vectors, strains and stiffnesses. Finally, we construct bases of rotationally symmetric subspaces to specify reduced parameter sets for symmetric fluctuation tensors.

Suppose the tensor space $\mathcal{V}^{n}$ is endowed with an orthogonal representation. Then, under the action (2.34) of the group $\mathit{SO}(3)$, the tensor space admits a harmonic decomposition [54]

3.1 $$ \mathcal{V}^{n} = \mathcal{D}^{n_{1}} \oplus \cdots \oplus \mathcal{D}^{n_{k}} $$

into $k$ subspaces $\mathcal{D}^{n_{j}}$ which are rotation-invariant and irreducible. The subspaces $\mathcal{D}^{n_{j}}$ are generally known as *irreducible* subspaces, and are each of tensor order $n_{j}$ and dimension ${(2n_{j}+1)}$, consisting of tensors which are fully symmetric and traceless. The harmonic decomposition (3.1) is not unique in the general case. To resolve this non-uniqueness, we define a convention to select a specific set of embeddings

3.2 $$ J^{\mathcal{V}}_{j}: \mathcal{D}^{n_{j}} \rightarrow \mathcal{V}^{n}. $$

Following from Eq. (3.1), the embeddings $J^{\mathcal{V}}_{j}$ are required to be pairwise orthogonal, meaning that for any tensors ${\mathbb{A}}^{n_{i}} \in \mathcal{D}^{n_{i}}$ and ${\mathbb{B}}^{n_{j}} \in \mathcal{D}^{n_{j}}$, we find

3.3 $$ J^{\mathcal{V}}_{i}( {\mathbb{A}}^{n_{i}} ) \cdot J^{\mathcal{V}}_{j}( {\mathbb{B}}^{n_{j}} ) = \delta _{ij} {\mathbb{A}}^{n_{i}} \cdot {\mathbb{B}}^{n_{j}} . $$

The embeddings allow the explicit and unique decomposition of any tensor ${\mathbb{V}}^{n} \in \mathcal{V}^{n}$ into a sum

3.4 $$ {\mathbb{V}}^{n} = \sum _{i=1}^{{{k}}} J^{\mathcal{V}}_{i}( {\mathbb{A}}^{n_{i}} _{i}), \quad {\mathbb{A}}^{n_{i}} _{i} \in \mathcal{D}^{n_{i}}. $$

To select a basis using embeddings, we also require orthonormal bases for the irreducible spaces

3.5 $$ \mathfrak{B}_{{\mathcal{D}}^{k}} = \left \{ {\mathbb{D}}^{k} _{j} \,\middle |\, j = \{1, \ldots , 2k+1 \}\right \}. $$

Here, we use the Z-axis convention, where the basis tensors ${\mathbb{D}}^{k} _{j}$ are eigenvectors of rotations around the Z-axis, with complex-valued eigenvectors recombined to yield basis tensors with real components [40]. Using the orthonormal bases of irreducible spaces together with a set of embeddings $\{J^{\mathcal{V}}_{i}\}$ (3.2), we choose the orthonormal harmonic basis

3.6 $$ \mathfrak{B}_{\mathcal{V}^{n}} = \left \{J^{\mathcal{V}}_{i}( {\mathbb{D}}^{n_{i}} _{j}) \,\middle |\, {\mathbb{D}}^{n_{i}} _{j} \in \mathfrak{B}_{\mathcal{D}^{n_{i}}} , i \in \{1, \ldots , k\} \right \} $$

of the space $\mathcal{V}^{n}$.

From the basis definition (3.6), an explicit method for selecting the embeddings (3.2) is missing. We wish for our harmonic bases (3.6) to reflect that our tensor spaces generally represent linear mappings. Therefore, we need to select embeddings for the dyadic product of the tensor space $\mathcal{V}^{n}$ with another tensor space $\mathcal{U}^{m}$. We denote the respective embeddings selected for the spaces $\mathcal{V}^{n}$ and $\mathcal{U}^{m}$ as in Eq. (3.2) and

3.7 $$ J^{\mathcal{U}}_{i} : \mathcal{D}^{m_{i}} \rightarrow \mathcal{U}^{m}. $$

We seek a basis of the product space

3.8 $$ \mathcal{W}^{m+n} = \mathcal{U}^{m} \otimes \mathcal{V}^{n}, $$

where the dyadic product ⊗ computes the additive closure of the set of all pairwise dyadic products of elements of $\mathcal{U}^{m}$ and $\mathcal{V}^{n}$, respectively. The resulting space $\mathcal{W}^{m+n}$ is naturally isomorphic with the space of linear mappings from $\mathcal{U}^{m}$ to $\mathcal{V}^{n}$. The Kronecker product × between embeddings is defined by the rule

3.9 $$\begin{aligned} J^{\mathcal{U}}_{i} \times J^{\mathcal{V}}_{j} &: \mathcal{D}^{n_{i}} \otimes \mathcal{D}^{n_{j}} \rightarrow \mathcal{U}^{m} \otimes \mathcal{V}^{n}, \\ (J^{\mathcal{U}}_{i} \times J^{\mathcal{V}}_{j})( {\mathbb{D}}^{m_{i}} _{i} \otimes {\mathbb{D}}^{n_{j}} _{j}) &= J^{\mathcal{U}}_{i}( {\mathbb{D}}^{m_{i}} _{i}) \otimes J^{\mathcal{V}}_{j}( {\mathbb{D}}^{n_{j}} _{j}), \end{aligned}$$

which yields a map from each deviatoric product space $( {\mathbb{D}}^{m_{i}} _{i} \otimes {\mathbb{D}}^{n_{j}} _{j})$ to the product space $\mathcal{W}^{m+n}$ (3.8). To proceed, it is required to select embeddings to facilitate a harmonic decomposition of dyadic products of deviatoric spaces. This is accomplished using the Clebsch-Gordan decomposition [55]

3.10 $$ \mathcal{D}^{m} \otimes \mathcal{D}^{n} = \sum _{k=|m-n|}^{{ {m+n}}} c^{mnk}(\mathcal{D}^{k}) $$

with the Clebsch-Gordan mappings

3.11 $$ c^{mnk}: \mathcal{D}^{k} \rightarrow \mathcal{D}^{m} \otimes \mathcal{D}^{n}, $$

that form a specific set of embeddings in the sense of Eq. (3.2). For their explicit computation, we refer to Krause et al. [40].

Using the Kronecker product of embeddings for $\mathcal{U}^{m}$ and $\mathcal{V}^{n}$ together with the Clebsch-Gordan decomposition (3.11), the harmonic basis of the product space $\mathcal{W}^{m+n}$ becomes

3.12 $$ \mathfrak{B}_{\mathcal{W}^{m+n}} = \left \{(J^{\mathcal{U}}_{i} \times J^{\mathcal{U}}_{j}) (c^{mn\ell _{i}}( {\mathbb{D}}^{\ell _{i}} _{k})) \,\middle |\, {\mathbb{D}}^{\ell _{i}} _{k} \in \mathfrak{B}_{\mathcal{D}^{\ell }_{k}} , k \in \{1, \ldots q \}\right \}. $$

This procedure allows us to iteratively select harmonic bases for successive products of tensor spaces.

### Representing the Fluctuation Tensor via a Harmonic Basis

The three-dimensional vector space  decomposes into a single irreducible subspace  under the action of the rotation group $\mathit{SO}(3)$. Following the $Z$-axis convention discussed above, the harmonic basis of the space  is chosen as the usual orthonormal basis 3.13 Applying the rule for bases of products (3.12), we write 3.14 where $\boldsymbol{1} $ is the second-order identity tensor and $\boldsymbol{\epsilon } $ denotes the third-order permutation or Levi-Civita tensor. Products between a tensor and a basis are applied to all basis tensors individually. The basis  of the zeroth-order space  contains only the scalar unity 1. In total, the resulting basis  contains nine pairwise orthogonal and normalized second-order tensors, which reflect the harmonic decomposition of the space  into the spherical, skew-symmetric and deviatoric subspaces.

Since stresses $\boldsymbol{\sigma } $ and strains $\boldsymbol{\varepsilon } $ are symmetric, we are interested in bases of symmetric subspaces. We define the transposition of a tensor ${\mathbb{W}}^{2m} \in \mathcal{U}^{m} \otimes \mathcal{U}^{m}$ by exchanging the respective basis tensors of each component, writing

3.15 $$ {\left ( {\mathbb{W}}^{2m} \right )}^{\mathsf{T}} = \sum _{ {\mathbb{U}}^{m} _{i} \in \mathfrak{B}_{\mathcal{U}^{m}} } \sum _{ {\mathbb{U}}^{m} _{j} \in \mathfrak{B}_{\mathcal{U}^{m}} } {\mathbb{U}}^{m} _{j} \otimes {\mathbb{U}}^{m} _{i} \left (\left ( {\mathbb{U}}^{m} _{i} \otimes {\mathbb{U}}^{m} _{j} \right ) \cdot {\mathbb{W}}^{2m} \right ). $$

Using this general transposition operation, we define the symmetrization operation

3.16 $$\begin{aligned} \operatorname{Sym} : \mathcal{U}^{m} \otimes \mathcal{U}^{m} \rightarrow \mathcal{U}^{m} \otimes \mathcal{U}^{m}, \end{aligned}$$

3.17 $$\begin{aligned} {\operatorname{Sym}}\left ( {\mathbb{W}}^{2m} \right ) = \frac{1}{2} \left ( {\mathbb{W}}^{2m} + \left ( {\mathbb{W}}^{2m} \right )^{\mathsf{T}}\right ). \end{aligned}$$

The basis of the symmetric subspace results from

3.18 $$ \mathfrak{B}_{{\operatorname{Sym}}\left ( \mathcal{U}^{m} \right )} = \operatorname{orthonormalize} \left (\left \{{\operatorname{Sym}} \left ( {\mathbb{W}}^{2m} \right ) \,\middle |\, {\mathbb{W}}^{2m} \in \mathfrak{B}_{\mathcal{U}^{m}} \right \}\right ), $$

where the orthonormalization operation is computationally accomplished by Gram-Schmidt orthogonalization [56]. The result of the Gram-Schmidt orthogonalization depends on the order of the input tensors. We impose a canonical ordering on our tensor bases which corresponds to the indices of the components respective to that basis. Gram-Schmidt orthogonalization with a specified order yields a unique result. Tensors which are zero after orthogonalization are discarded.

For the second-order symmetric space , the harmonic basis reads 3.19 This basis separates hydrostatic and shear modes, as evidenced by the strain components

3.20 $$ \boldsymbol{\varepsilon } \hat{=} \begin{pmatrix} \frac{1}{\sqrt{3}} \left (\varepsilon _{11} + \varepsilon _{22} + \varepsilon _{33}\right ) \\ \frac{1}{\sqrt{2}} (\varepsilon _{11} - \varepsilon _{22}) \\ \sqrt{2} \varepsilon _{12} \\ \sqrt{2} \varepsilon _{13} \\ \sqrt{2} \varepsilon _{23} \\ \frac{1}{\sqrt{6}}\left (2 \varepsilon _{33} - \varepsilon _{11} - \varepsilon _{22}\right ) \end{pmatrix} \textstyle\begin{array}{r @{\quad}l} \left . \right \} & \text{{{hydrostatic}} (spherical)}\\ \left . \textstyle\begin{array}{c} \\ \\ \\ \\ \\ \end{array}\displaystyle \right \} & \text{shear (deviatoric)} \end{array}\displaystyle . $$

The hydrostatic-shear-separation implies a representation of stiffness tensors as block matrices 
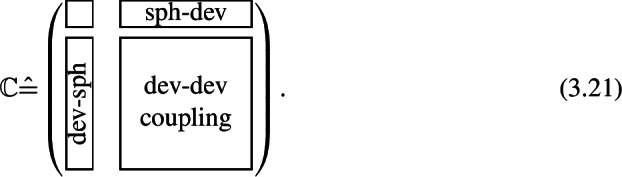
 To find a matrix representation of the eighth-order fluctuation tensor, we require a harmonic basis of the stiffness space . We apply the general basis formula (3.18) to harmonically decompose the stiffness into 3.22 where the tensors $A$ are elements of the irreducible subspaces $\mathcal{D}^{k}$ of appropriate order ${k \in \{0,2,4\}}$, specifically ${A^{\circ }, A^{ {\prime } } \in \mathcal{D}^{0}}$, , and ${ {\mathbb{A}} ^{ {\prime \prime \prime } } \in \mathcal{D}^{4}}$. We use the symbols ′ and ∘ as indices to directly associate the terms with their physical meaning. The associated embeddings apply as

3.23 $$\begin{aligned} J^{\circ }(A^{\circ }) &= A^{\circ } {\mathbb{P}} ^{\circ }, \end{aligned}$$

3.24

3.25 $$\begin{aligned} J^{ {\prime } }(A^{ {\prime } }) &= \frac{A^{ {\prime } }}{\sqrt{5}} {\mathbb{P}} ', \end{aligned}$$

3.26 $$\begin{aligned} J^{ {\prime \prime } }( \boldsymbol{A} ^{ {\prime \prime } }) &= \sqrt{\frac{3}{7}} \, {\mathbb{P}} '( \boldsymbol{1} \times \boldsymbol{A} ^{ {\prime \prime } } + \boldsymbol{A} ^{ {\prime \prime } } \times \boldsymbol{1} ) {\mathbb{P}} ', \end{aligned}$$

3.27 $$\begin{aligned} J^{ {\prime \prime \prime } }({ {\mathbb{A}} }^{ {\prime \prime \prime } }) &= { {\mathbb{A}} }^{ { \prime \prime \prime } }, \end{aligned}$$

using the spherical projector ${\mathbb{P}} ^{\circ }$ and the deviatoric projector ${\mathbb{P}} '$. In Eq. (3.26), the symbol × denotes the Kronecker product between even-order tensors

3.28 $$ {{\left ( \boldsymbol{A} \times \boldsymbol{B} \right )_{ijkl} = A_{ik} B_{jl}.}} $$

As even-order tensors are linear maps, their Kronecker products are defined analogously to the case of embeddings (3.9), such that they obey the rule

3.29 $$ {{( \boldsymbol{A} \times \boldsymbol{B} )( \boldsymbol{C} ) = \boldsymbol{A} \boldsymbol{C} \boldsymbol{B} ^{\mathsf{T}}.}} $$

Closely inspecting the various embeddings reveals that Eq. (3.22) is equivalent to the well-known harmonic decomposition of the stiffness [34, 57].

Using the fourth-order 21-dimensional harmonic basis, we write the eighth-order fluctuation tensor as a matrix 
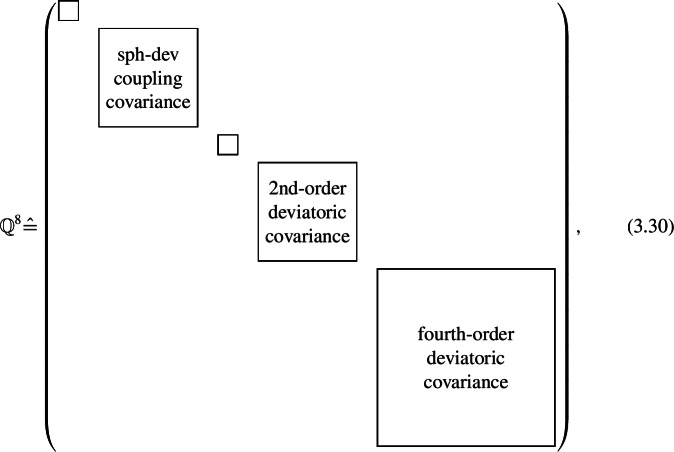
 with a block structure induced by the harmonic decomposition of the stiffness (3.22). Each block contains the fluctuation of the stiffness components corresponding to one irreducible subspace. The small non-labeled blocks are the variance of the bulk modulus $K$ and the shear modulus $G$ respectively. The various non-diagonal blocks contain fluctuation terms corresponding to covariances between different subspaces. These blocks are omitted in Eq. (3.30) for clarity and are not generally zero.

The symmetric basis formula (3.18) defines an eighth-order basis of the fluctuation tensor space . This 231-dimensional basis  is useful particularly for symmetry considerations. We compute the eighth-order harmonic decomposition and find that a number of product subspaces appear multiple times, with different embeddings. For example, since the stiffness space  contains two second-order deviatoric subspaces $\mathcal{D}^{2}$ with respective embeddings  and , the product space  contains four subspaces $\mathcal{D}^{2}\otimes \mathcal{D}^{2}$ with four different embeddings given by Kronecker products (3.9). After symmetrization, three product combinations, i.e., three different embeddings of the deviator space product ${\mathcal{D}^{2} \otimes \mathcal{D}^{2}}$, remain. To obtain deviatoric subspaces, we apply the Clebsch-Gordan decomposition (3.11), which decomposes the product ${\mathcal{D}^{2} \otimes \mathcal{D}^{2}}$ into the subspaces ${\mathcal{D}^{0}}$, ${\mathcal{D}^{2}}$ and ${\mathcal{D}^{4}}$. Odd-order subspaces are not present since the fluctuation tensor space has major index symmetry. Applying this procedure to all products results in the total number of irreducible subspaces listed in Table 1.

**Table 1:** Multiplicity of harmonic subspaces of

Product subspace	Harmonic subspaces				
	$\mathcal{D}^{0}$	$\mathcal{D}^{2}$	$\mathcal{D}^{4}$	$\mathcal{D}^{6}$	$\mathcal{D}^{8}$
$\mathcal{D}^{0} \otimes \mathcal{D}^{0}$	3				
$\mathcal{D}^{2} \otimes \mathcal{D}^{2}$	3	3	3		
$\mathcal{D}^{4} \otimes \mathcal{D}^{4}$	1	1	1	1	1
${\operatorname{Sym}}\left ( \mathcal{D}^{2} \otimes \mathcal{D}^{0} \right )$		4			
${\operatorname{Sym}}\left ( \mathcal{D}^{4} \otimes \mathcal{D}^{0} \right )$			2		
${\operatorname{Sym}}\left ( \mathcal{D}^{4} \otimes \mathcal{D}^{2} \right )$		2	2	2	
Total	7	10	8	3	1

So far, stiffness and fluctuation tensor harmonic bases remained somewhat abstract. To further illustrate the various components, we introduce a visualization method based on Böhlke and Brüggemann’s visualization [36] of arbitrarily anisotropic stiffness tensors as two polar plots whose distances to the origin are given as functions of the normal vector $\boldsymbol{n} \in S_{2}$ by the direction-dependent Young’s modulus $E( \boldsymbol{n} )$

3.31 $$ E( \boldsymbol{n} ) = \frac{1}{\left ( \boldsymbol{n} \otimes \boldsymbol{n} \right ) \cdot \mathbb{C} ^{-1} [ \boldsymbol{n} \otimes \boldsymbol{n} ]} $$

and bulk modulus

3.32 $$ K^{*}( \boldsymbol{n} ) = \frac{1}{\left ( \boldsymbol{n} \otimes \boldsymbol{n} \right ) \cdot \mathbb{C} ^{-1} [\frac{1}{3} \boldsymbol{1} ]}. $$

Instead of considering the compliance $\mathbb{C} ^{-1}$, we directly visualize the stiffness tensor, defining the bulk modulus as

3.33 $$ K( \boldsymbol{n} ) = \frac{1}{3} \left ( \boldsymbol{n} \otimes \boldsymbol{n} \right ) \cdot \mathbb{C} [ \boldsymbol{1} ]. $$

Instead of the Young’s modulus $E( \boldsymbol{n} )$, we use the direction-dependent shear modulus

3.34 $$ G( \boldsymbol{n} ) = \frac{1}{6} \left (3 \boldsymbol{n} \otimes \boldsymbol{n} - \boldsymbol{1} \right ) \cdot \mathbb{C} [3 \boldsymbol{n} \otimes \boldsymbol{n} - \boldsymbol{1} ]. $$

**Fig. 1:**
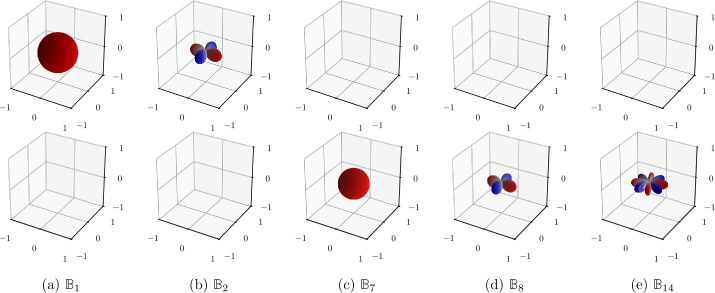
Example basis tensors of the minor- and major-symmetric fourth-order harmonic basis , visualized as hydrostatic (top) and shear (bottom) figures. From left to right, figures show examples from pure hydrostatic, hydrostatic-shear coupling, and the three irreducible shear subspaces. Red values are positive, blue are negative

The shear modulus contains all data of the shear-shear block in the block matrix stiffness (3.21), whereas the bulk modulus encodes the characteristics of the hydrostatic-hydrostatic block and the coupled shear-hydrostatic block. Therefore, by plotting both quantities, all data defining an arbitrarily anisotropic stiffness tensor is encoded in two images. Example tensors of the basis  are visualized in Fig. 1. Each tensor is visualized as a spherical harmonic function of the same order as its irreducible subspace.

Similar direct visualizations of the eighth-order tensor ${\mathbb{Q}}^{8} $ are more challenging. Unlike the two plots required for stiffness visualization, a total of ten plots would be needed, since ten is the highest multiplicity of irreducible subspaces in Table 1. Instead of printing ten plots per tensor, we employ the visualizations of stiffness anisotropy modes to explain the components of the block matrix form (3.30) as variances and covariances of anisotropy stiffness modes.

### Symmetries of the Fluctuation Tensor

In general, as the symmetric $21\times 21$ matrix representation (3.30) shows, the fluctuation tensor has

3.35 $$ \sum _{k=1}^{21} k = \frac{21 \cdot 22}{2} = 231 $$

independent components, which can be reduced further in case rotational symmetries are present. Fluctuation tensors which are invariant with respect to a rotation group $S \subseteq \mathit{SO}(3)$ (2.39) form a linear subspace. The orthogonal projection onto an $S $-symmetric tensor subspace of arbitrary order $n$ is defined by the integral

3.36 $$ P_{ S }( {\mathbb{V}}^{n} ) = \int _{ S } \boldsymbol{R} \star {\mathbb{V}}^{n} {\,\mathrm{d}}{V( \boldsymbol{R} )}, $$

where ${\,\mathrm{d}}{V}$ stands for the normalized Haar measure on the symmetry group $S $. The deviatoric subspaces comprising the harmonic decomposition of a tensor space (3.1) are each closed under rotation, meaning that a rotated deviatoric tensor is always deviatoric. Therefore, a tensor rotation (2.34) of a harmonically decomposed tensor ${\mathbb{V}}^{n} $ (3.4) applies to each of the deviatoric tensors ${ {\mathbb{A}}^{n_{i}} _{i} \in \mathcal{D}^{n_{i}}_{i}}$ separately via

3.37 $$ \boldsymbol{R} \star {\mathbb{V}}^{n} = \sum _{i=1}^{k} J^{\mathcal{V}}_{i}( \boldsymbol{R} \star {\mathbb{A}}^{n_{i}} _{i}), \quad \boldsymbol{R} \in \mathit{SO}(3). $$

The orthogonal projection (3.36) computes as

3.38 $$ P_{S}( {\mathbb{V}}^{n} ) = \sum _{i=1}^{k} J^{\mathcal{V}}_{i}(P_{ S }( {\mathbb{A}}^{n_{i}} _{i})). $$

Consequently, when modeling symmetries of tensors of any order, it is sufficient to discuss symmetries of irreducible spaces. In Table 2, the dimensions of common rotationally symmetric irreducible spaces are listed. We computed these dimensions explicitly as the matrix rank of the respective projections $P_{ S }$. Computation approaches without using projection matrices are well-known in classical representation theory [58], as detailed for a similar example to ours by Man [38, example 16.18]. Combined with Table 1, the number of independent parameters of the fluctuation tensor follows directly as the sum of dimensions of all involved symmetric irreducible spaces. As an example, for isotropic symmetry, only the zeroth-order subspace $\mathcal{D}^{0}$ does not vanish, with dimension 1 remaining according to Table 2. Since it occurs seven times in total in the fluctuation tensor space  according to Table 1, isotropic fluctuation tensors have seven independent parameters.

**Table 2:** Dimension of rotationally symmetric irreducible spaces

Symmetry	Deviatoric spaces				
	$\mathcal{D}^{0}$	$\mathcal{D}^{2}$	$\mathcal{D}^{4}$	$\mathcal{D}^{6}$	$\mathcal{D}^{8}$
Isotropic	1	0	0	0	0
Cubic	1	0	1	0	1
Transversal-isotropic	1	1	1	1	1
Orthotropic	1	2	3	4	5
Fully anisotropic	1	5	9	13	17

For rotationally symmetric irreducible spaces, we find bases by symmetrization and orthonormalization

3.39 $$ \mathfrak{B}_{P_{S}(\mathcal{D}^{k})} = \operatorname{orthonormalize} \left (\left \{P_{S}( {\mathbb{D}}^{k} _{i}) \,\middle |\, i \in \{1, \ldots , 2k+1\}\right \}\right ). $$

Corresponding rotationally symmetric harmonic bases follow from the definition of a harmonic basis (3.6) as

3.40 $$ \mathfrak{B}_{\mathcal{V}^{n}} = \left \{I_{i}( {\mathbb{D}}^{n_{i}} _{j}) \,\middle |\, {\mathbb{D}}^{n_{i}} _{j} \in \mathfrak{B}_{S(\mathcal{D}^{n_{i}})} , i \in \{1, \ldots k \}\right \}. $$

We outline the computation for the case of isotropy. Following Table 2, the only isotropic irreducible space is the zeroth-order space  with the basis given by 3.41 In the harmonic decomposition of the product space ${ \mathcal{C} \otimes \mathcal{C} }$, we find the zeroth-order space seven times, as noted in Table 1. In the Clebsch-Gordan decomposition (3.10), zeroth-order subspaces arise only from the product of irreducible subspaces of equal tensor order. Therefore, to compute the required embeddings, we combine all pairs of same-order stiffness subspace embeddings (3.23) via the Kronecker product (3.9) with the Clebsch-Gordan mappings (3.11). Symmetrization yields embeddings from the isotropic irreducible space  to the fluctuation space, which we apply to the basis tensor of the space , i.e., the number 1. After normalizing, we acquire the seven isotropic basis tensors 3.42 Using the $21\times 21$ matrix form (3.30), an isotropic fluctuation tensor appears as 3.43 where the symbol $I_{d}$ denotes a $d\times d$ identity matrix. In Eq. (3.43), the components $Q^{\circ }$, , $Q^{ {\prime } }$, $Q^{ {\prime \prime } }$ and $Q^{ {\prime \prime \prime } }$ have physical meaning as variances for the various block matrices of the matrix form of the stiffness (3.21). None of the variance terms vanishes. Consequently, isotropy of the fluctuation tensor does not preclude fluctuations of anisotropic stiffness terms. However, due to the vanishing off-diagonal terms in the identity matrices, individual anisotropic fluctuations within each block are uncorrelated. The components $Q^{\circ {\prime } }$ and  quantify correlations between blocks of equal order.

As an example, the component $Q^{\circ }$ quantifies the variance of the bulk modulus. A correlation of the bulk modulus $K$ with any of the anisotropic blocks is forbidden by fluctuation tensor isotropy. A correlation between the bulk modulus $K$ and the shear modulus $G$ is allowed, and quantified by the component $Q^{ \circ {\prime } }$.

To further understand what isotropy of the fluctuation tensor implies, we refer to the anisotropic modes illustrated in Fig. 1. There are zeroth- and second-order modes in the hydrostatic diagram, and modes of order zero, two and four in the shear diagram. An overview of covariance components as related to those modes is given in Table 3. Each of the components $Q^{\circ }$, , $Q^{ {\prime } }$, $Q^{ {\prime \prime } }$ and $Q^{ {\prime \prime \prime } }$ encodes the variance of modes in either the shear and hydrostatic diagram. Modes of the same order within the same diagram always have the same variance, so that only five scalars are required to describe all variances. The components $Q^{\circ {\prime } }$ and  similarly quantify covariances between the same modes in shear and hydrostatic diagrams, leading to a total of seven scalars. All other covariances vanish, such as those between modes of mixed orders or different modes of the same order.

**Table 3:** List of isotropic fluctuation tensor components in terms of covariances of stiffness modes

Component	$Q^{\circ }$		$Q^{ {\prime } }$	$Q^{ {\prime \prime } }$	$Q^{ {\prime \prime \prime } }$	$Q^{\circ {\prime } }$	
Covariance of modes	${\mathbb{B}} _{1}$, ${\mathbb{B}} _{1}$	${\mathbb{B}} _{1+i}$, ${\mathbb{B}} _{1+i}$ *i*∈{1…5}	${\mathbb{B}} _{7}$, ${\mathbb{B}} _{7}$	${\mathbb{B}} _{7+i}$, ${\mathbb{B}} _{7+i}$ *i*∈{1…5}	${\mathbb{B}} _{12+i}$, ${\mathbb{B}} _{12+i}$ *i*∈{1…9}	${\mathbb{B}} _{1}$, ${\mathbb{B}} _{7}$	${\mathbb{B}} _{1+i}$, ${\mathbb{B}} _{7+i}$ *i*∈{1…5}

We move on to the cubic fluctuation tensor. In addition to the seven isotropic components, the cubic fluctuation tensor contains ten anisotropic cubic components, which we denote by the symbols $Q_{8}$ to $Q_{17}$. These cubic components are somewhat more complicated in their definition than the isotropic components. Each of the irreducible spaces $\mathcal{D}^{4}$, $\mathcal{D}^{6}$ and $\mathcal{D}^{8}$ contains one cubic basis tensor, denoted ${\mathbb{D}}^{n} _{ {\mathrm{cub}} }$ respectively. Consequently, we require all products of harmonic stiffness subspaces which involve fourth, sixth or eighth-order deviatoric terms. We identify these via the Clebsch-Gordan decomposition (3.10). After combining the Clebsch-Gordan mappings (3.11) with the appropriate stiffness subspace embeddings (3.23), we acquire the anisotropic cubic fluctuation tensor basis tensors 3.44 With the isotropic and cubic basis tensors, we directly compute the symmetry projections

3.45 $$\begin{aligned} P_{\mathrm{iso}}( {\mathbb{T}}^{8} ) = \sum _{i=1}^{7} {\mathbb{B}}^{8} _{i} \left ( {\mathbb{T}}^{8} \cdot {\mathbb{B}}^{8} _{i}\right ) \end{aligned}$$

and

3.46 $$\begin{aligned} P_{\mathrm{cub}}( {\mathbb{T}}^{8} ) = \sum _{i=1}^{17} {\mathbb{B}}^{8} _{i} \left ( {\mathbb{T}}^{8} \cdot {\mathbb{B}}^{8} _{i}\right ), \end{aligned}$$

where the isotropic basis tensors are enumerated from ${\mathbb{B}}^{8} _{1}$ to ${\mathbb{B}}^{8} _{7}$ for simplicity of notation.

The above results can be extended to arbitrary symmetry groups in a straightforward manner: First, the symmetric basis tensors of the deviatoric spaces must be identified. Then, the embeddings may be used to compute basis tensors of the harmonically decomposed space of interest. The results are quite lengthy to write down for arbitrary symmetries and may obfuscate the message of the paper at hand. Therefore, we restrict ourselves to the cubic and the isotropic case. Production processes without a directional bias result in statistically isotropic ensembles. Periodizing an isotropic ensemble with a cubic volume element yields an ensemble with cubic symmetry, as discussed in Sect. 2.3. Therefore, a large subset of practical examples is covered by the given formulas.

## Computational Investigations

### Computing the Fluctuation Tensor

We compute the apparent linear elastic properties using a fast Fourier transform (FFT) based homogenization approach [11]. All computations take place on a voxel-based grid representation of the microstructure volume, which is a periodic cube of edge length $L$. The balance of linear momentum (2.14) is discretized using the rotated staggered grid approach [59]. The resulting equation system is solved using the conjugate gradient method [60, 61]. By prescribing six linearly independent effective strains, six apparent stresses are obtained, fully characterizing the apparent stiffness $\mathbb{C} ^{\mathrm{app}} _{{ { L }}}$ in $(6\times 6)$ matrix form.

For each heterogeneous material under consideration, we randomly generate $N$ microstructure cells to sample the ensemble. In this setting, the periodized ensemble average of the apparent stiffness (2.18) is estimated by the random tensor

4.1 $$ \langle \mathbb{C} ^{\mathrm{app}} _{{ { L }}}\rangle _{N} = \frac{1}{N} \sum _{i=1}^{N} \mathbb{C} ^{\mathrm{app}} _{{ { L }}, i}. $$

The unbiased covariance estimator for the apparent stiffness [62]

4.2 $$ \langle \mathbb{C} ^{\mathrm{app}} _{{ { L }}} \otimes \mathbb{C} ^{\mathrm{app}} _{{ { L }}}\rangle _{N} - \langle \mathbb{C} ^{\mathrm{app}} _{{ { L }}}\rangle _{N}\otimes \langle \mathbb{C} ^{\mathrm{app}} _{{ { L }}}\rangle _{N} = \frac{1}{N-1} \sum _{i=1}^{N} \left ( \mathbb{C} ^{\mathrm{app}} _{{ { L }}, i} \otimes \mathbb{C} ^{\mathrm{app}} _{{ { L }}, i} - \langle \mathbb{C} ^{\mathrm{app}} _{{ { L }}}\rangle _{N}\otimes \langle \mathbb{C} ^{\mathrm{app}} _{{ { L }}}\rangle _{N}\right ) $$

leads to an estimated apparent fluctuation tensor (2.26) of

4.3 $$ {\mathbb{Q}}^{8} _{ L ,N} = \frac{L^{3}}{N-1} \sum _{i=1}^{N} \left ( \mathbb{C} ^{\mathrm{app}} _{{ { L }}, i} \otimes \mathbb{C} ^{\mathrm{app}} _{{ { L }}, i} - \langle \mathbb{C} ^{\mathrm{app}} _{{ { L }}}\rangle _{N}\otimes \langle \mathbb{C} ^{\mathrm{app}} _{{ { L }}}\rangle _{N}\right ). $$

To interpret the accuracy of the estimated fluctuation tensor with respect to the sample size $N$, we compute the standard deviation of apparent fluctuation tensor components. We assume that the apparent stiffness $\mathbb{C} ^{\mathrm{app}} $ is normally distributed, based on results obtained in the limit ${L\rightarrow \infty}$ by Duerinckx et al. [30] for thermal conductivity. Consequently, the $(21\times 21)$ matrix of estimated fluctuation components 4.4 follows a Wishart distribution $\mathcal{W}( \boldsymbol{V} , N)$ [63, 64] with the positive-definite $(21\times 21)$ scale matrix $\boldsymbol{V} $. The expectation of each component is given by [64]

4.5 $$ E(Q_{ L ,N,ij}) = N V_{ij}. $$

We assume that the expectation of the component $Q_{ L ,N,ij}$ is close to the estimated value from numerical computation to retrieve

4.6 $$ V_{ij} \approx \frac{1}{N} Q_{ L ,N,ij}. $$

The standard deviation of the component $Q_{ L ,N,ij}$ is given by [64]

4.7 $$\begin{aligned} \operatorname{std}\left (Q_{ L ,N,ij}\right ) &= N \left (V_{ij}^{2} + V_{ii} V_{jj}\right ) \\ &\approx \frac{1}{N} \left (Q_{ L ,N,ij}^{2} + Q_{ L ,N,ii} Q_{ L ,N,jj} \right ). \end{aligned}$$

The estimate for the standard deviation (4.7) rests on the assumption that the stiffness $\mathbb{C} ^{\mathrm{app}} $ is normally distributed, which is not generally true for volumes of finite size. Therefore, the estimate represents a heuristic approximation which serves as an indicator of uncertainty, not a rigorous quantification.

Since the apparent fluctuation tensor components are computed using sums of random samples, i.e., a Monte-Carlo method, the standard deviation can also be estimated using the Monte-Carlo convergence rate. Asymptotically, the error scales as

4.8 $$ \operatorname{err} = \mathcal{O}\left (\frac{1}{\sqrt{N}}\right ), $$

from which it follows that the standard deviations for different sample sizes $M$ and $N$ are related by

4.9 $$ \operatorname{std}\left ( {\mathbb{Q}}^{8} _{ L ,M}\right ) = \sqrt{\frac{N}{M}} \operatorname{std}\left ( {\mathbb{Q}}^{8} _{ L ,N}\right ) $$

as the sample sizes $N$ and $M$ tend towards infinity. For a finite set of $N$ samples, we choose $N/M$ disjoint subsets of $M$ samples each. We estimate the standard deviation of the subset fluctuation tensors ${\mathbb{Q}}^{8} _{ L ,M,i}$ directly using the bias-free estimator

4.10 $$ \operatorname{std}\left ( {\mathbb{Q}}^{8} _{ L ,M}\right ) \approx \sqrt{\frac{1}{N/M-1} \sum _{i=1}^{N/M} ( {\mathbb{Q}}^{8} _{ L ,M,i}- {\mathbb{Q}}^{8} _{ L ,N})^{2}}, $$

where all operations are understood to be component-wise. Since the standard deviation in Eq. (4.10) is estimated using a sample standard deviation, a large number of samples $N/M$ is required. However, the subset fluctuation tensors ${\mathbb{Q}}^{8} _{ L ,M,i}$ are also based on covariances estimated from a sample standard deviation (4.2), thus requiring a large subset size $M$ for accuracy.

Using Eq. (4.9), we estimate the standard deviation for the full sample size $N$ as

4.11 $$ \operatorname{std}\left ( {\mathbb{Q}}^{8} _{ L ,N}\right ) \approx \sqrt{\frac{N}{N-M} \sum _{i=1}^{N/M} ( {\mathbb{Q}}^{8} _{ L ,M,i}- {\mathbb{Q}}^{8} _{ L ,N})^{2}}. $$

The approximation (4.11) involves an additional source of error for finite sample sizes $M$ and $N$, since Eq. (4.9) holds in the limit of infinite sample and subset sample sizes.

Unlike the direct standard deviation estimate, the Monte-Carlo approach does not assume a normal probability distribution. Therefore, it can be used to estimate the standard deviation of arbitrary expressions of fluctuation tensor components, such as the norm of the fluctuation tensor. We provide both estimates when possible.

As discussed in Sect. 2, statistical symmetries constrain the fluctuation tensor. These properties can be used to validate our results by employing the symmetry projectors $P_{\mathrm{iso}}$ (3.45) and $P_{ {\mathrm{cub}} }$ (3.46). We consider statistically isotropic ensembles, such that the apparent fluctuation tensor for a fixed length $L$ is of cubic symmetry (2.46). Computing the estimated apparent fluctuation tensor ${\mathbb{Q}}^{8} _{ L ,N}$ (4.3) from a finite number of realizations $N$ leads to further randomness, which is arbitrarily anisotropic in general. Consequently, we define the relative anisotropic remainder

4.12 $$ e_{Q,\mathrm{aniso}} = \frac{\| {\mathbb{Q}}^{8} _{ L ,N} - P_{ {\mathrm{cub}} }( {\mathbb{Q}}^{8} _{ L ,N}) \|}{\| P_{ {\mathrm{cub}} }( {\mathbb{Q}}^{8} _{ L ,N}) \|}. $$

In the limit of infinitely many realizations, the estimated apparent fluctuation tensor converges to the apparent fluctuation tensor by the law of large numbers, leading to the error convergence

4.13 $$ e_{Q,\mathrm{aniso}} \longrightarrow 0 \quad \mathrm{as} \quad N \longrightarrow \infty . $$

For a sufficiently large number of realizations $N$, the anisotropic remainder is expected to conform to the Monte-Carlo convergence rate [30]

4.14 $$ e_{Q,\mathrm{aniso}} \lesssim N^{-\frac{1}{2}}. $$

Assuming that the randomness induced by the finite number of realizations $N$ is approximately unbiased with regard to its effect on isotropic, cubic and anisotropic parts of the apparent fluctuation tensor, we can use the anisotropic remainder $e_{Q,\mathrm{aniso}}$ to validate the accuracy of our results with regard to the chosen number of realizations $N$.

A similar symmetry consideration can be used to quantify convergence of the apparent fluctuation tensor ${\mathbb{Q}}^{8} _{ L }$ towards the fluctuation tensor ${\mathbb{Q}}^{8} $. We define the relative cubic remainder

4.15 $$ e_{Q,\mathrm{cub}} = \frac{\| {\mathbb{Q}}^{8} _{ L ,N} - P_{\mathrm{iso}}( {\mathbb{Q}}^{8} _{ L ,N}) \|}{\|P_{\mathrm{iso}}( {\mathbb{Q}}^{8} _{ L ,N})\| }. $$

Isotropy of the fluctuation tensor implies

4.16 $$ e_{Q,\mathrm{cub}} \longrightarrow 0 \quad \mathrm{as} \quad L \longrightarrow \infty . $$

For a sufficiently large volume element edge-length $L$, the cubic remainder is expected to conform to the theoretically obtained convergence rate [30]

4.17 $$ e_{Q,\mathrm{cub}} \lesssim L^{-\frac{3}{2}} $$

up to logarithmic terms.

### Spherical e-Glass Inclusions

We consider microstructures consisting of E-glass spheres embedded in a polyamide matrix. The linear elastic stiffness of both phases is given in Table 4.

**Table 4:** Young’s modulus $E$ and Poisson ratio $\nu $ for E-glass and polyamide [65]

	*E* in GPa	*ν*
E-glass	72.0	0.22
Polyamide	2.1	0.3

**Fig. 2:**
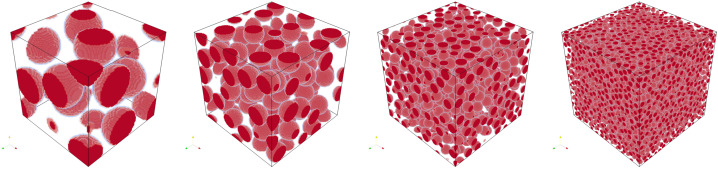
Example microstructures with lengths from 128 μm to 1024 μm, doubling at each step

To generate periodic microstructures, we adapt the Sequential Addition and Migration (SAM) algorithm [50]. The SAM algorithm was designed for short fiber inclusions, which are modeled as spherocylinders. We set the fiber length to zero, such that the spherocylinders degenerate to spheres. The SAM algorithm adds a small number of spheres at random positions, moves the spheres to resolve collisions, and repeats the prior steps until the desired volume fraction of inclusions is reached. We consider spheres with a uniform diameter of 50 μm and a total volume fraction of 30%. A minimum distance of 10 μm between spheres is imposed by virtually enlarging the sphere diameter to 60 μm during collision evaluations. Example microstructures with edge lengths from 128 μm to 1024 μm are shown in Fig. 2. Since the initial sphere candidate positions are chosen randomly, the microstructure generation algorithm samples a random periodic ensemble for each microstructure size [51]. We assume that, as the edge length tends towards infinity, the sequence of periodicized ensembles converges to an ensemble which is ergodic and statistically isotropic. When computing effective properties, we characterize this ensemble.

Since the effective properties cannot be computed directly, we compute reference values based on microstructures with an edge length of $1024~\upmu \mbox{m}$. We choose a resolution of 2 μm per voxel, thus resolving the minimum sphere distance by five voxels. The effective stiffness (4.1) and the apparent fluctuation tensor (4.3) are computed based on 2000 random realizations. As the effective stiffness is isotropic (2.42), we characterize it using the bulk modulus

4.18 $$\begin{aligned} \langle K^{ \mathrm{app} }\rangle _{N,L} = 12{,}797.0~\mbox{MPa}, \quad \operatorname{std}\left (K^{ \mathrm{app} }\right ) _{N,L} = 0.8~\mbox{MPa} \end{aligned}$$

and shear modulus

4.19 $$\begin{aligned} \langle G^{ \mathrm{app} }\rangle _{N,L} = 36{,}214.6~\mbox{MPa}, \quad \operatorname{std}\left ({{G}}^{ \mathrm{app} }\right ) _{N,L} = 4.0~\mbox{MPa}, \end{aligned}$$

where the standard deviations are given by the bias-free estimator from the sample standard deviation (4.2). Consequently, the isotropic elastic constant standard deviations are related to the estimated fluctuation tensor via Eq. (4.3). For the bulk and shear moduli, we retrieve the scalar equations

4.20 $$ \operatorname{std}\left (K^{ \mathrm{app} }_{L}\right ) = \frac{1}{3} \sqrt{\frac{Q^{\circ }}{L^{3}}} \quad \mathrm{and} \quad \operatorname{std}\left (G^{ \mathrm{app} }_{L}\right ) = \frac{1}{2} \sqrt{\frac{Q^{ {\prime } }}{L^{3}}}. $$

Notably, the variances which are immediately accessible from isotropic constants account for only two components $Q^{\circ }$ and $Q^{ {\prime } }$ of the seven-component isotropic fluctuation tensor.

We compute all seven fluctuation tensor components using the same set of data as the stiffness values, resulting in Table 5. The standard deviations of the fluctuation tensor components are shown to indicate the degree of confidence in the results. Two different methods were used to compute the standard deviations, a Monte-Carlo approximation (4.11) using the subset sample size $M=10$ and an approximation based on the Wishart distribution (4.7). Both methods yield similar results. Based on either result, the standard deviation is less than 5% for any component. The hydrostatic and shear variance terms $Q^{\circ }$ and $Q^{ {\prime } }$ are two orders of magnitude smaller than the largest term, the fourth-order deviatoric term $Q^{ {\prime \prime \prime } }$. To put this discrepancy into context, we compute stiffness component standard deviations for the largest investigated volumes with side length $L=1024~\upmu \mbox{m}$. Quantitatively, the stiffness component standard deviation implied by the largest term $Q^{ {\prime \prime \prime } }$ equals 45 MPa, whereas the standard deviation implied by the largest scalar fluctuation term $Q^{ {\prime } }$ only amounts to 7.9 MPa. Taking only scalar variances into account therefore neglects the larger part of the stiffness fluctuations. For uncertainty quantification, a tensorial representation of fluctuations is necessary.

**Table 5:** Fluctuation tensor components of the glass-sphere microstructure with respect to the isotropic basis tensors (3.42), computed using microstructures with side length ${L=1024~\upmu \mbox{m}}$. Standard deviations indicate uncertainty due to the limited number of realizations ${N=2000}$

Component	$Q^{\circ }$		$Q^{ {\prime } }$	$Q^{ {\prime \prime } }$	$Q^{ {\prime \prime \prime } }$	$Q^{\circ {\prime } }$	
Value in MPa^2^ mm^3^	6.5	339.0	67.1	294.1	2158.0	27.2	436.8
MC std (4.11) in MPa^2^ mm^3^	0.2	4.7	2.1	4.3	20.1	0.9	6.3
Relative MC std in %	3.1	1.4	3.1	1.4	0.9	3.2	1.4
Wishart std (4.7) in MPa^2^ mm^3^	0.2	4.8	2.1	4.2	22.8	0.8	5.4
Relative Wishart std in %	3.2	1.4	3.2	1.4	1.1	2.8	1.2

**Fig. 3:**
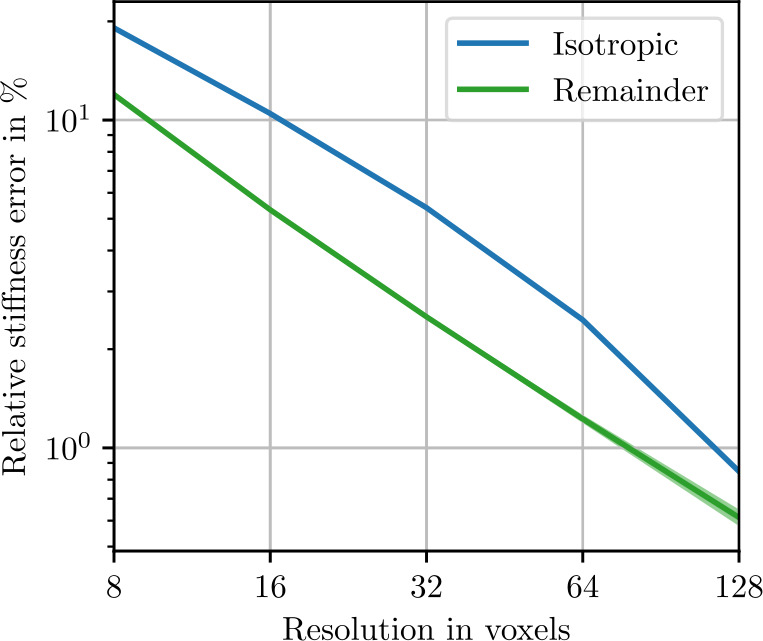
Stiffness resolution study with ${N=1000}$ realizations of the sphere inclusion ensemble with edge length 128 μm. Shaded areas indicate twice the standard deviation (4.11) of the respective stiffness norms

To control the influence of the number of voxels chosen to discretize the microstructure, we perform a resolution study. Using a small microstructure of edge length 128 μm, we compute the expected apparent stiffness (4.1) and the apparent fluctuation tensor (4.3) with $N=1000$ realizations each. In Fig. 3, the isotropic stiffness error

4.21 $$\begin{aligned} e_{\mathrm{iso},\mathrm{res}} &= \frac{\| P_{\mathrm{iso}}( \mathbb{C} _{L,N,\mathrm{res}}) - P_{\mathrm{iso}}( \mathbb{C} _{L,N,256})\|}{\| \mathbb{C} _{L,N,256}\|} \end{aligned}$$

relative to a reference stiffness computed with a resolution of 256 voxels per edge is shown along with the anisotropic remainder

4.22 $$\begin{aligned} e_{\mathrm{iso},\mathrm{res}} &= \frac{\| \mathbb{C} _{L,N,\mathrm{res}} - P_{\mathrm{iso}}( \mathbb{C} _{L,N,\mathrm{res}})\|}{\| \mathbb{C} _{L,N,256}\|}. \end{aligned}$$

**Fig. 4:**
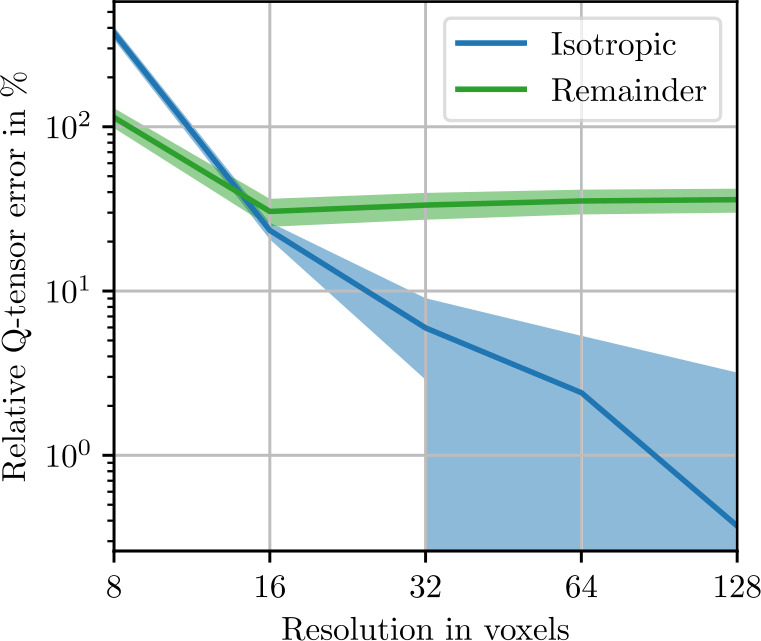
Fluctuation tensor resolution study with ${N=1000}$ realizations of the sphere inclusion ensemble with edge length 128 μm. Shaded areas indicate twice the standard deviation (4.11) of the respective fluctuation tensor norms

Both the isotropic and anisotropic errors decrease from more than 10% to less than % with increasing resolution. Standard deviations, computed with a Monte-Carlo approach (4.11), are very small, indicating high confidence for these results. In Fig. 4, the same data set is used to compute errors for the fluctuation tensor. The errors are numerically larger and show higher standard deviations. Additionally, the anisotropic remainder plateaus at a resolution of 16 voxels along the microstructure edge. This remaining anisotropic error is therefore not due to insufficient resolution. In the following, we use 2 μm per voxel, which corresponds to 64 voxels in the figures.

**Fig. 5:**
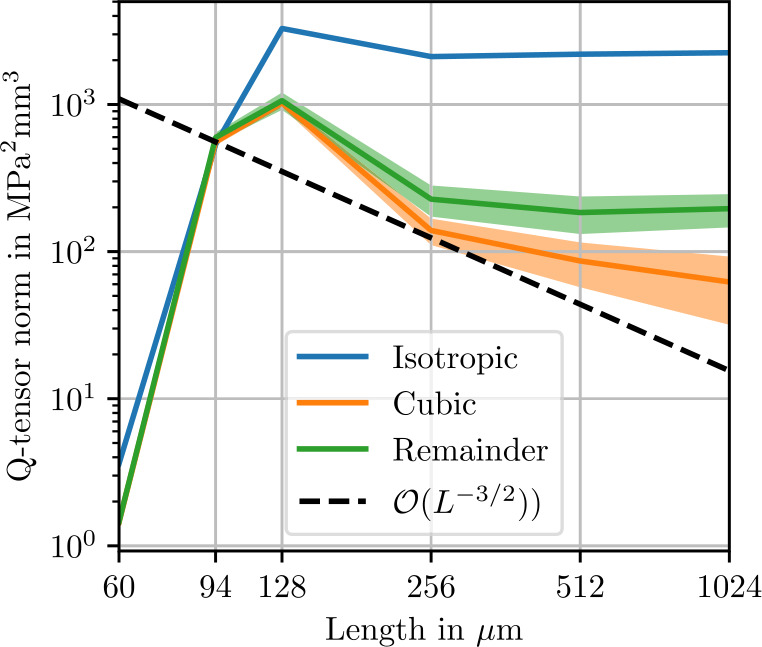
Isotropic, cubic and anisotropic norms of the estimated apparent fluctuation tensor (4.3) of the sphere inclusion ensemble. Values computed from 2000 realizations each. Shaded areas indicate twice the standard deviation (4.11) of the respective stiffness norms

**Fig. 6:**
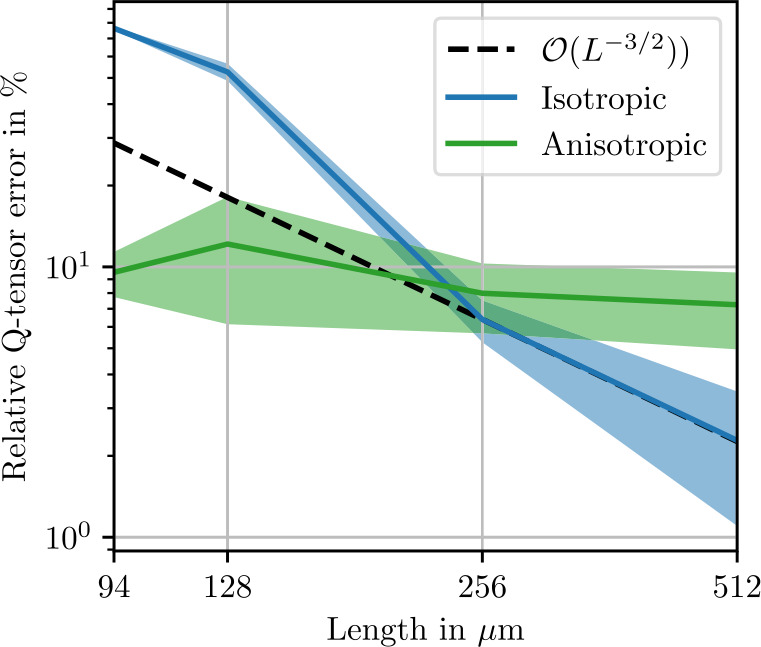
Error in the isotropic components of the fluctuation tensor computed using a reference of 2000 realizations of length $L=1024~\upmu \mbox{m}$. Values computed from 2000 realizations each. Shaded areas indicate twice the standard deviation (4.11) of the respective stiffness norms

Figure 5 shows norms of the isotropic, cubic and anisotropic parts of the apparent fluctuation tensor for 2000 realizations each of differently-sized microstructures. The smallest volume elements of 60 μm contain only one sphere. In theory, these volume elements all represent the same periodic cubic lattice of spherical inclusion. Without fluctuations in the microstructure geometry, the comparatively small stiffness covariance must be due to discretization errors. In the volume element with a length of 94 μm, four spheres are present, marking a sharp transition to a noticeable level of stiffness covariance. As the volume element size increases further, the norm of the isotropic part of the fluctuation tensor converges to roughly $3\times10^{3}~\mbox{MPa}^{2}\,\mbox{mm}$. In Fig. 6, the error in the isotropic components of the apparent fluctuation tensor is computed using the microstructure of length 1024 μm as a reference. The expected theoretical convergence rate (2.28) is approximately reached. Returning to Fig. 5, the cubic remainder, which is expected to converge to zero at the rate (4.17) of $\mathcal{O}(L^{-3})$, does not reach this rate at high resolutions. This observation is presumably a numerical artifact due to an insufficient number of realizations, causing random fluctuations of the cubic components of the fluctuation tensor, which translate to a non-zero mean norm of the cubic part. Similarly, the anisotropic remainder (4.12), which should vanish due to symmetry, is non-zero in the norm due to an insufficient number of realizations.

**Fig. 7:**
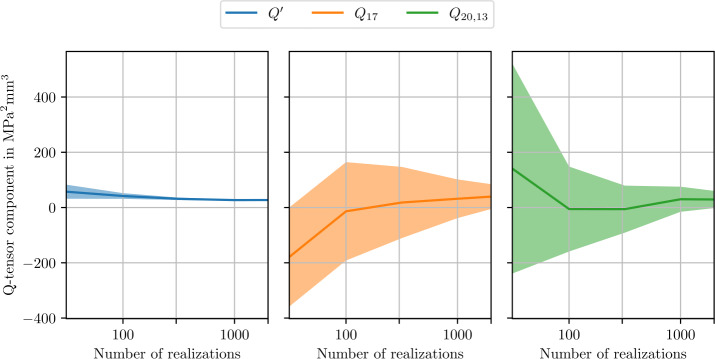
Selected isotropic, cubic and anisotropic components of the estimated apparent fluctuation tensor (4.3) for varying numbers of realizations of length $L=512~\upmu \mbox{m}$. Shaded areas indicate twice the standard deviation (4.11) of the respective components

To quantify the influence of the number of realizations, we plot components of the fluctuation tensor directly. We select one isotropic, one cubic and one fully anisotropic component and show their convergence in Fig. 7. The isotropic component converges to a non-zero value, while the anisotropic component is indistinguishable from zero. For the depicted microstructure of length 512 μm, the cubic component is strongly influenced by remaining fluctuations. These fluctuations arise due to using a limited number of realizations and are responsible for the lower convergence rate of the cubic component in Fig. 5.

Our observed convergence rates are similar to those theoretically derived for thermal conductivity [30]. Compared to effective stiffness computation, computing fluctuation tensors accurately requires a large sample size and involves higher levels of noise. Enforcing isotropy of the fluctuation tensor using the projection (3.45) is a useful post-processing technique, as it filters out sizable anisotropic noise.

### Fiber-Reinforced Polymers

**Fig. 8:**
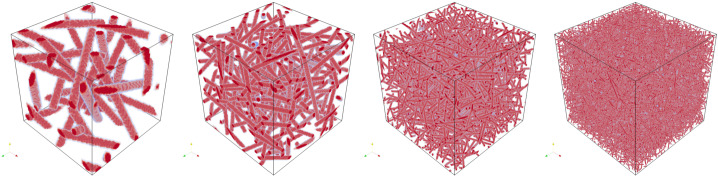
Example microstructures with volume element edge lengths from 128 μm to 1024 μm, doubling at each step

We move on to E-glass fibers in a polymer matrix. The material properties are the same as in the prior example, given in Table 4. We assume cylindrical fibers of length 250 μm and diameter 10 μm with a total fiber volume fraction of 10%. To generate microstructures, we again use the SAM algorithm [50]. We prescribe a minimum distance of 4 μm between fibers, as well as an isotropic fourth-order fiber orientation tensor [66]. Example microstructures of various sizes are shown in Fig. 8.

**Fig. 9:**
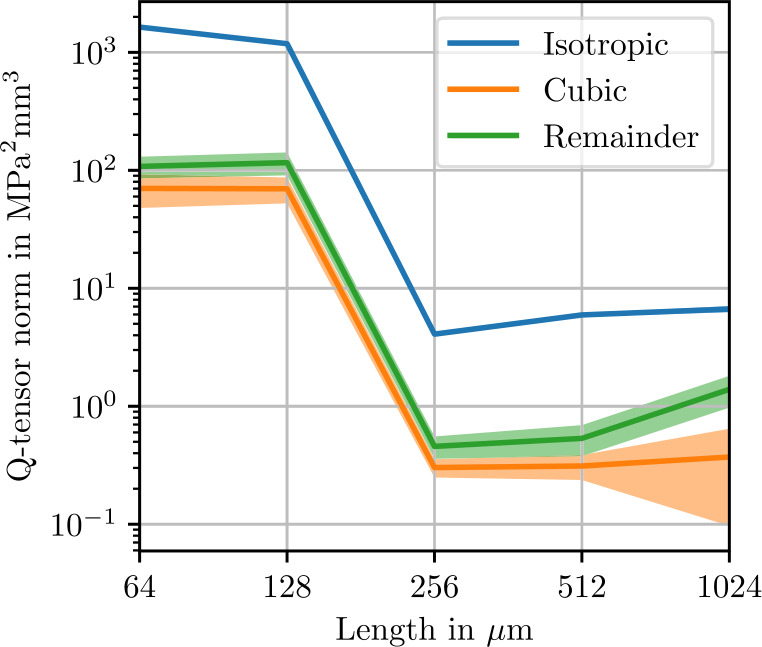
Isotropic, cubic and anisotropic norms of the estimated apparent fluctuation tensor (4.3) computed from 1000 realizations each of the short-fiber reinforced ensemble. Shaded areas indicate twice the standard deviation (4.11) of the respective stiffness norms

**Fig. 10:**
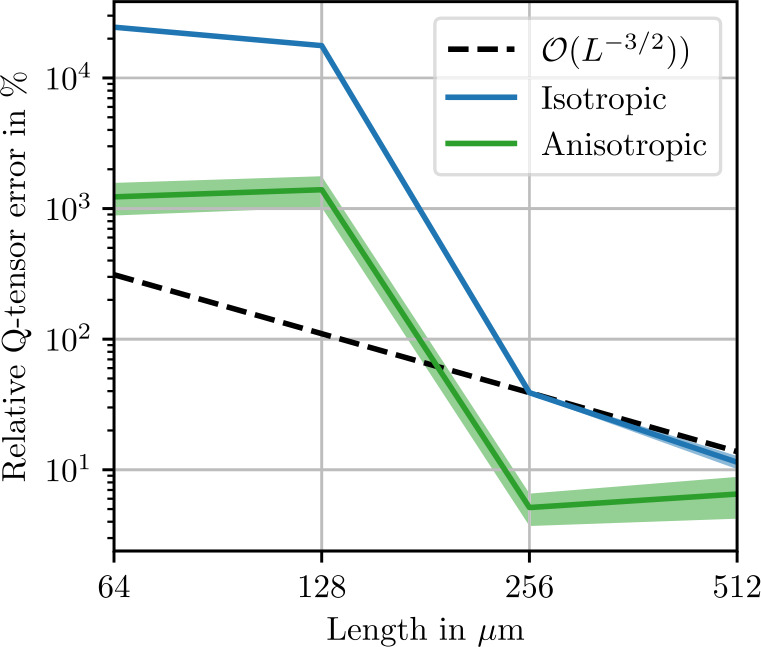
Error in the isotropic components of the fluctuation tensor computed using a reference of 1000 realizations of length $L=1024~\upmu \mbox{m}$. Shaded areas indicate twice the standard deviation (4.11) of the respective stiffness norms

Norms of the isotropic, cubic and anisotropic remainder of the fluctuation tensor are shown in Fig. 9. All norms decrease by two orders of magnitude going from a cell edge length of 128 μm to 256 μm, which is likely related to the fiber length of 250 μm. Beyond this edge length, the theoretical convergence rate (2.28) is reached, as illustrated in Fig. 10. We conclude that for fluctuation tensor computation, it is not recommended to use microstructures which are smaller than the fiber length, but microstructures need not be much larger to yield accurate results. Even more so than with the sphere microstructures, the cubic remainder is dominated by the anisotropic remainder, such that the theoretical convergence rate is not evident in the cubic term in Fig. 9.

In Table 6, the isotropic components of the short-fiber reinforced ensemble are listed. Unlike the sphere-inclusion ensemble, the fiber ensemble fluctuation tensor has equal orders of magnitude across all components. Generally, hydrostatic fluctuations are higher than pure shear fluctuations, with the largest fluctuation found in the second-order hydrostatic-deviator coupling $Q_{2}$. As observed in the sphere-inclusion ensemble, higher-order components have higher fluctations, with the fourth-order deviatoric fluctuation $Q_{5}$ being twice as large as the zeroth-order deviatoric fluctuation $Q_{0}$. Again, the covariances $Q_{6}$ and $Q_{7}$ are roughly as large as the individual components, showing that stiffness fluctuations are strongly correlated. A microstructure that is stiff with respect to spherical deformation modes tends to also be stiff with respect to same-order deviatoric deformation modes.

**Table 6:** Fluctuation tensor components of the short-fiber-reinforced microstructure with respect to the isotropic basis tensors (3.42). Computed using the microstructure of length $L=1024$ μm

Component	$Q^{\circ }$		$Q^{ {\prime } }$	$Q^{ {\prime \prime } }$	$Q^{ {\prime \prime \prime } }$	$Q^{\circ {\prime } }$	
Value in MPa^2^ mm^3^	2.9	4.4	1.1	1.4	2.1	1.8	2.4
MC std (4.11) in MPa^2^ mm^3^	0.23	0.16	0.04	0.05	0.07	0.17	0.10
Relative MC std in %	7.9	3.6	4.1	3.3	3.4	9.5	4.1
Wishart std (4.7) in MPa^2^ mm^3^	0.29	0.20	0.11	0.06	0.07	0.18	0.11
Relative Wishart std in %	10.0	4.5	10.0	4.5	3.4	10.0	4.5

### Polycrystalline Copper

We turn to polycrystalline copper as an example for a common polycrystalline material with pronounced single-crystal elastic anisotropy. We generate polycrystalline microstructures via centroidal Laguerre tessellations [48], using the open-source python module crystallites [67], which is available at https://git.uni-due.de/publicsoftwareingmath/crystallites/. The software crystallites implements optimization algorithms for the crystallite orientations to match prescribed texture coefficients [68]. To avoid an influence on the fluctuation tensor results, we do not use these optimization algorithms for our baseline study. Instead, the orientations are sampled randomly using no further optimization.

**Fig. 11:**
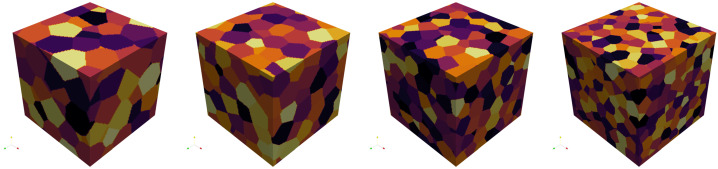
Example microstructures containing from 64 to 512 grains, doubling at each step. Colors indicate the first Euler angle of each grain orientation

We study microstructures comprised of equally-sized grains with an equivalent diameter of 50 μm. Example microstructures with different numbers of grains are shown in Fig. 11. All microstructures are discretized with $2^{14}$ voxels per grain, which is more than sufficient for linear elastic polycrystal homogenization [67]. For the material model, we assume linear elasticity with cubic single crystal elastic constants as given in Table 7.

**Table 7:** Single crystal elastic constants of copper [69]

${C_{1111}}$	${C_{1122}}$	${C_{1212}}$
170.2 GPa	114.9 GPa	61.0 GPa

**Fig. 12:**
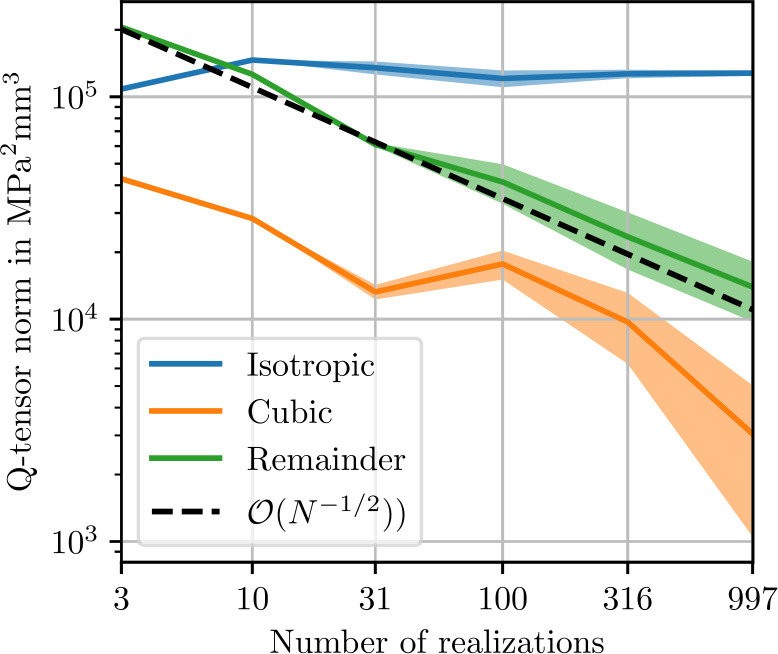
Isotropic fluctuation tensor norm and cubic and anisotropic remainders for increasing numbers of realizations of a 1024-grain microstructure. Shaded areas indicate twice the standard deviation (4.11) of the respective stiffness norms

In Fig. 12, the fluctuation tensor norms are shown for an increasing number of realizations. The anisotropic remainder (4.12) closely follows the expected convergence rate (4.14). The cubic remainder follows the same convergence rate, and can therefore be considered a consequence of anisotropic error, as opposed to error stemming from the periodization of the ensemble, which would not vary with the number of realizations.

**Fig. 13:**
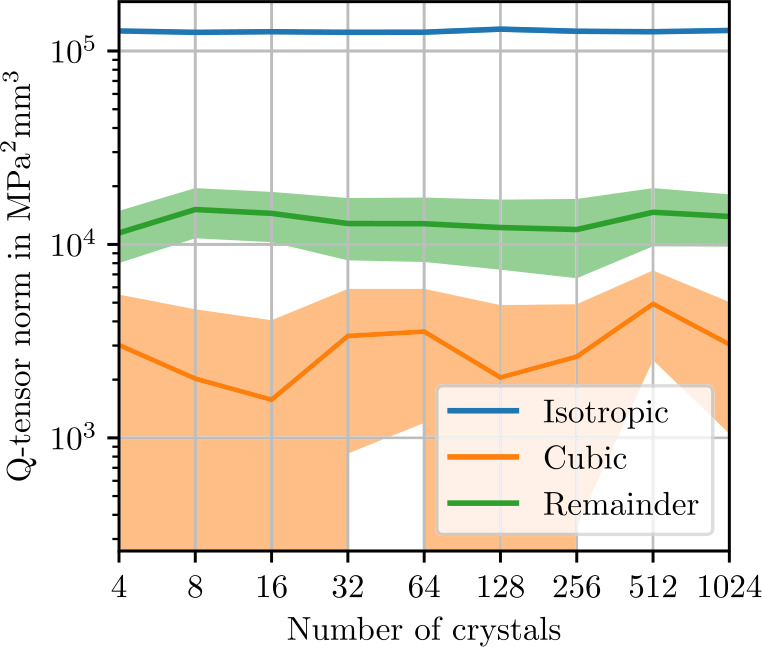
Fluctuation tensor norm and anisotropic remainders for microstructures with increasing numbers of grains, computed with 1000 realizations each. Shaded areas indicate twice the standard deviation (4.11) of the respective stiffness norms

**Fig. 14:**
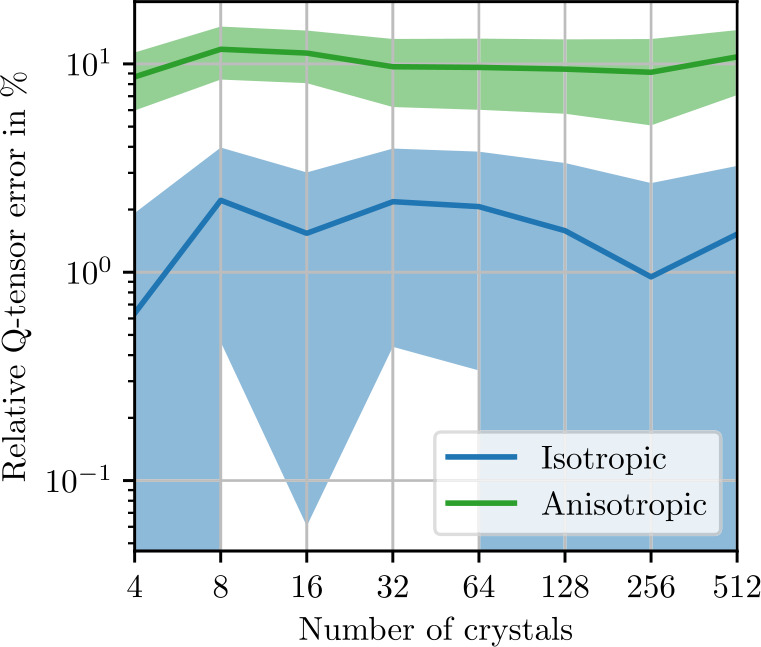
Relative fluctuation tensor error and anisotropic remainders for microstructures with increasing numbers of grains, computed with 1000 realizations each. The reference value is based on 1000 realizations of a 1024 grain microstructure. Shaded areas indicate twice the standard deviation (4.11) of the respective stiffness norms

We study the convergence behavior of the apparent fluctuation tensor with increasingly large microstructures, as parametrized by an increasing number of grains. In Fig. 13, fluctuation tensor norms are plotted for an increasing number of grains spanning the range from four to 1024 grains. We observe no meaningful change in the isotropic fluctuation tensor norm over this range. For the relative isotropic error depicted in Fig. 14, the theoretical convergence rate is not observed. As the relative isotropic error is dominated by the anisotropic remainder (4.12), we conclude that the error due to an insufficient number of realizations exceeds the error due to an insufficient number of grains even for microstructures comprised of only four grains.

**Fig. 15:**
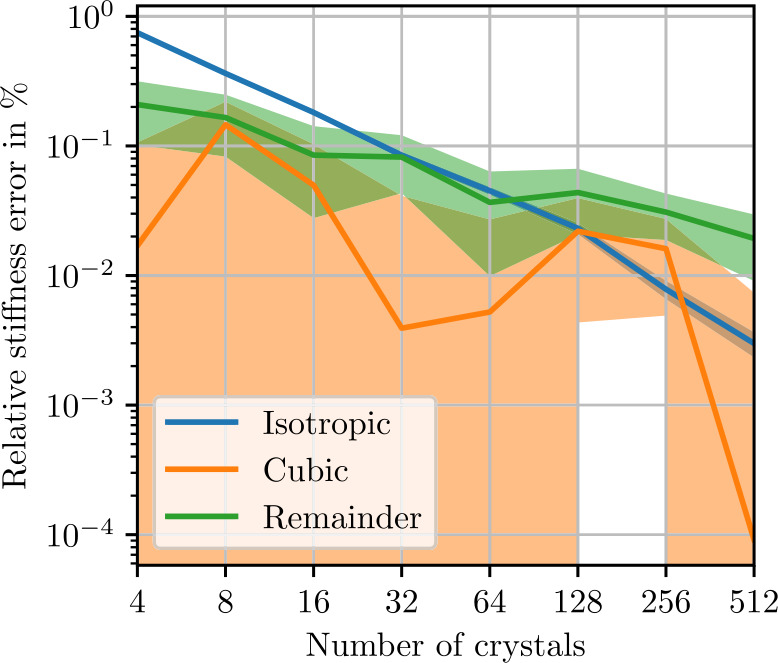
Relative mean stiffness error and anisotropic remainders for microstructures with increasing numbers of grains, computed with 1000 realizations each. The reference value is based on 1000 realizations of a 1024 grain microstructure. Shaded areas indicate twice the standard deviation (4.11) of the respective stiffness norms

By contrast, the stiffness is strongly affected by the number of grains. Using the same stiffness data, we compute the mean stiffness of 1000 realizations $\langle \mathbb{C} ^{\mathrm{app}} \rangle _{1000, L }$ (4.1) as an estimate of the effective stiffness. As shown in Fig. 15, the error in the computed isotropic properties decreases with an increasing number of grains. Unlike in the fluctuation tensor computations, the anisotropic remainder also slightly declines with the microstructure size. Comparing the values of the respective error, we note that the stiffness is accurate to within % even for microstructures consisting of only four grains, as is the fluctuation tensor.

We conclude that, while effective stiffness computation for a polycrystal microstructure is made more accurate by using large RVEs, accurate fluctuation tensor computation instead demands very large numbers of realizations for further accuracy. If an accuracy to within 1% is sufficient, very small RVEs suffice for either quantity.

In Table 8, the isotropic components of the fluctuation tensor of the polycrystal ensemble are listed. All stiffness components involving cubic parts of the stiffness block matrix (3.21) are constants, since the compressive stiffness of a single-phase cubic polycrystal is homogeneous. Correspondingly, the fluctuation components $Q^{\circ }$, , $Q^{\circ {\prime } }$ and , which involve hydrostatic terms, must be zero. Of the pure shear components $Q^{ {\prime } }$, $Q^{ {\prime \prime } }$ and $Q^{ {\prime \prime \prime } }$, the term $Q^{ {\prime \prime \prime } }$ dominates by three orders of magnitude, similarly to the sphere-reinforced microstructure in Table 5. Since the fiber microstructure does not show a similarly outsized influence of the term $Q^{ {\prime \prime \prime } }$, it initially appears that microstructures with approximately spherical morphologies have large fourth-order deviatoric fluctuations $Q^{ {\prime \prime \prime } }$. However, this morphological explanation does not hold in the following example.

**Table 8:** Fluctuation tensor components of a 512 grain microstructure with respect to the isotropic basis tensors (3.42)

Component	$Q^{\circ }$		$Q^{ {\prime } }$	$Q^{ {\prime \prime } }$	$Q^{ {\prime \prime \prime } }$	$Q^{\circ {\prime } }$	
Value in MPa^2^ mm^3^	0.0	0.0	293	679	125,744	0.0	0.0
MC std (4.11) in MPa^2^ mm^3^	0.0	0.0	13	12	1810	0.0	0.0
Relative MC std in %			4.3	1.7	1.4		
Wishart std (4.7) in MPa^2^ mm^3^	0.0	0.0	13	14	1874	0.0	0.0
Relative Wishart std in %			4.5	2.0	1.5	3.2	1.4

The stiffness variance of polycrystal microstructures can be reduced by prescribing texture coefficient tensors for each generated volume element [68]. Using this technique, volume elements of a given size are more representative of the ensemble, which leads to faster convergence of the apparent stiffness [67].

We study the effect of this variance reduction technique on the fluctuation tensor of the statistically isotropic copper microstructure by prescribing vanishing texture coefficient tensors up to eighth order. The isotropic fluctuation tensor components for 1000 realizations of a microstructure comprising 256 grains are listed in Table 9. Compared to the microstructure without prescribed texture coefficients, the component $Q^{ {\prime \prime \prime } }$ is two orders of magnitude smaller, while the components $Q^{ {\prime } }$ and $Q^{ {\prime \prime } }$ are slightly increased. We observe that the difference in fluctuation tensors is substantial, considering that the effective shear moduli of both ensembles take the statistically indistinguishable values

4.23 $$\begin{aligned} \bar{G}_{\mathrm{random}} &= 100.19~\mbox{GPa}, \quad \operatorname{std}\left (\bar{G}_{\mathrm{random}}\right ) = 0.14~\mbox{GPa}, \end{aligned}$$

4.24 $$\begin{aligned} \bar{G}_{\mathrm{iso8}} &= 100.17~\mbox{GPa}, \quad \operatorname{std}\left (\bar{G}_{\mathrm{iso8}}\right ) = 0.14~\mbox{GPa}. \end{aligned}$$

This example shows that variance reduction techniques which do not introduce systematic errors in the effective stiffness may nonetheless significantly influence the fluctuation tensor. Additionally, the polycrystal microstructure with prescribed texture tensors does not have the large fourth-order deviatoric fluctuations $Q^{ {\prime \prime \prime } }$ associated with both random polycrystals and spherical inclusions in prior examples. Indeed, prescribing the texture tensors yields deviatoric fluctuation tensor components more reminiscent of the fiber microstructure in their relative magnitudes. We conclude that the dependence of stiffness fluctuation tensor components on microstructure statistics is complex and cannot be reduced to simple morphological heuristics.

**Table 9:** Fluctuation tensor components of a 256 grain microstructure with prescribed isotropic texture coefficient tensors up to eighth order, given with respect to the isotropic basis tensors (3.42)

Component	$Q^{\circ }$		$Q^{ {\prime } }$	$Q^{ {\prime \prime } }$	$Q^{ {\prime \prime \prime } }$	$Q^{\circ {\prime } }$	
Value in MPa^2^ mm^3^	0.0	0.0	640	1394	1876	0.0	0.0
MC std (4.11) in MPa^2^ mm^3^	0.0	0.0	27	24	31	0.0	0.0
Relative MC std in %			4.0	1.7	1.5		
Wishart std (4.7) in MPa^2^ mm^3^	0.0	0.0	30	27	30	0.0	0.0
Relative Wishart std in %			4.5	2.0	1.5		

Since variance reduction techniques significantly influence fluctuation tensor values, their use must be carefully considered. In the polycrystal case, randomly choosing the grain orientations can be motivated by the physical mechanism of grains randomly crystallizing out of molten metal, whereas prescribing texture tensors for each finite volume element is a numerical optimization that cannot be physically motivated. Therefore, we recommend using randomly sampled orientations for fluctuation tensor computations.

## Conclusion

This work introduces the elastic eighth-order fluctuation tensor as a natural tensor-valued characteristic used for quantifying apparent stiffness fluctuations of non-representative volume elements. We derive symmetry properties of the fluctuation tensor, establish a concise representation, and compute the fluctuation tensor for statistically isotropic sphere, fiber and polycrystal microstructures. We do not have a mathematical proof for the convergence properties of the fluctuation tensor at hand. Our computational results, however, show that the fluctuation tensor converges with the rates expected from analogy to heat conductivity [30], both with respect to the volume element size and the number of realizations.

The fluctuation tensor can be accurately computed using FFT-based computational homogenization methods. Both the volume element size and the number of realizations are critical to fluctuation tensor accuracy. To ascertain that either parameter is sufficiently large, we introduce a technique to decompose the fluctuation tensor of a statistically isotropic ensemble into an isotropic part, a cubic and an anisotropic remainder. In theory, the cubic remainder allows estimating the volume element size convergence, whereas the anisotropic remainder allows estimating convergence in the number of realizations. The anisotropic remainder proved a reliable convergence estimator for the considered microstructures. However, the cubic remainder was generally dominated by the anisotropic remainder, making it unsuitable. In practice, we therefore recommend comparing the isotropic components to a high-resolution reference value to ascertain fluctuation tensor convergence with respect to volume element size. The decomposition of the fluctuation tensor by symmetry also serves as an error mitigation technique. Discarding the anisotropic parts of the fluctuation tensor reduces errors due to small volume elements and small numbers of realizations. The discussed examples assume statistical isotropy, but extension to other statistical symmetries is straightforward using the eighth-order harmonic basis introduced in this work.

The fluctuation tensor quantifies stiffness fluctuations by taking into account the full tensorial nature of eighth-order covariances. Unlike a component-wise scalar fluctuation, this tensor includes non-obvious terms involving isotropic fluctuations of anisotropic stiffness terms, which dominate the fluctuation tensor for the spherical inclusion and randomly oriented polycrystal examples. Consequently, we recommend using the full fluctuation tensor for quantifying the random stiffness error, as opposed to scalar fluctuations of isotropic stiffness parameters. Since the fluctuation tensor is linked to the stiffness covariance, it may serve as the input for random models of stiffness fields which require the full tensorial stiffness covariance [41].

We also investigated the influence of variance reduction techniques, i.e., ensuring that each VE individually matches statistical properties of the entire ensemble. Since these techniques are intended to not induce additional bias in effective property computation, the magnitude of their effect on fluctuation tensor computation was not initially clear. Specifically, the fluctuation tensor is a property of the underlying stochastic ensemble, which we define only implicitly as the limit of a sequence of periodized ensembles with a periodic length tending to infinity. The effect of a variance reduction technique based on matching statistical properties vanishes in the limit of infinite VEs, since the infinite VE is statistically identical to the ensemble due to ergodicity. For effective properties, this works well in practice, and no bias is observed when matching statistical properties as diverse as volume fractions [18] and orientation tensors [67]. However, computing the fluctuations requires the apparent property variances, which also vanish in the limit of infinite VEs. In the example of polycrystals, prescribing isotropic orientation tensors strongly reduces the fluctuations of the anisotropic part of the stiffness. The same anisotropy terms are also small for the fiber microstructure example. We suspect that the underlying isotropization effect is similar despite the fundamental differences between prescribed fiber orientation tensors, which quantify morphological texture, and prescribed polycrystal orientation tensors, which quantify crystallographic texture. While further investigations are necessary, our findings suggest that variance reduction techniques need to be employed with special care in fluctuation tensor computations, as also suggested by Jeulin et al. [25] in the context of integral range computations.

## Data Availability

All data is available on request.

## Appendix: Symmetries of Apparent Properties

We show that statistical symmetry of the ensemble implies statistical symmetry of linear elastic properties, using the approach employed by Nguyen et al. [33] for thermal conductivity. Symmetry (2.39) of a probability measure $\mu _{ L }$ respective to the action of a rotation (2.37) requires

A.1 $$ \boldsymbol{R} \star \mu _{ L }= \mu _{ L }\quad \text{for any} \quad \boldsymbol{R} \in S _{ L } . $$

Consequently, for any function  which is integrable with respect to the measure $\mu _{ L }$, statistical symmetry implies

A.2 $$ \int _{\mathcal{C}_{{{ L }}}} F( \mathbb{C} ) {\,\mathrm{d}}{\mu _{ L }( \mathbb{C} )} = \int _{\mathcal{C}} F( \mathbb{C} ) {\,\mathrm{d}}{( \boldsymbol{R} \star \mu _{ L })( \mathbb{C} )} \quad \text{for all rotations} \quad \boldsymbol{R} \in S _{ L }. $$

With a change of variables, we write

A.3 $$\begin{aligned} \int _{\mathcal{C}_{{{ L }}}} F( \mathbb{C} ) {\,\mathrm{d}}{( \boldsymbol{R} \star \mu _{ L })( \mathbb{C} )} &= \int _{ \mathcal{C}} F( \mathbb{C} ) {\, \mathrm{d}}{\mu _{ L }( \boldsymbol{R} \star \mathbb{C} )} \\ &= \int _{\mathcal{C}_{{{ L }}}} F({ \boldsymbol{R} }^{\mathsf{T}} \star \tilde {\mathbb{C}} ) {\,\mathrm{d}}{ \mu _{ L }(\tilde {\mathbb{C}} )}. \end{aligned}$$

We define the homogenization function  which maps a periodic stiffness field to its apparent stiffness (2.18)

A.4 $$ H( \mathbb{C} ) = \mathbb{C} ^{\mathrm{app}} . $$

Since the balance of linear momentum (2.14) is rotation-invariant and the periodic boundary conditions (2.15) to (2.16) obey the periodic domain symmetry $S _{Y_{L}}$, the homogenization function $H$ has periodic symmetry

A.5 $$ \boldsymbol{R} \star H = H \quad \text{for all} \quad \boldsymbol{R} \in S _{\mu _{L}}. $$

Applying this symmetry to Eq. (A.3), we find

A.6 $$\begin{aligned} \int _{\mathcal{C}_{{{ L }}}} H({ \boldsymbol{R} }^{\mathsf{T}} \star \tilde {\mathbb{C}} ) {\,\mathrm{d}}{ \mu _{ L }(\tilde {\mathbb{C}} )} = \int _{\mathcal{C}} \boldsymbol{R} \star H(\tilde {\mathbb{C}} ) {\, \mathrm{d}}{\mu _{ L }( \tilde {\mathbb{C}} )}, \end{aligned}$$

from which we conclude

A.7 $$\begin{aligned} \boldsymbol{R} \star \langle \mathbb{C} ^{\mathrm{app}} \rangle _{L} = \langle \mathbb{C} ^{\mathrm{app}} \rangle _{L} \quad \text{for all} \quad \boldsymbol{R} \in S _{ L }. \end{aligned}$$

Therefore, statistical symmetry implies apparent stiffness symmetry with respect to the periodic stiffness group.

For the sake of completeness, we discuss the symmetry of the effective stiffness. We assume a domain $Y_{D}$ which is not cubic, but spherical. We impose vanishing Dirichlet conditions

A.8 $$ \boldsymbol{u} ( {\boldsymbol{x}} ) = \boldsymbol{0} \quad \text{for all} \quad {\boldsymbol{x}} \in \partial Y_{D} $$

on the domain boundary $\partial Y_{D}$. An apparent stiffness with the usual properties also follows in this setting [21]. Since the spherical domain is isotropic

A.9 $$ S _{Y_{D}} = \mathit{SO}(3), $$

the periodic symmetry group (2.43) simplifies to

A.10 $$ S _{\mu _{D}} = S _{\mu}. $$

Consequently, the apparent stiffness in this setting has the statistical symmetry of the ensemble, not the reduced symmetry of the periodized ensemble. Since this ensemble-symmetric apparent stiffness also converges to the effective stiffness, the effective stiffness must reflect the statistical symmetry of the ensemble. The symmetry of the effective stiffness can also be proven without using the apparent stiffness [14, 42].

Next, we discuss symmetries of the apparent fluctuation tensor. We define the second-moment homogenization function  as

A.11 $$\begin{aligned} H^{2}( \mathbb{C} ) = \mathbb{C} ^{\mathrm{app}} \otimes \mathbb{C} ^{\mathrm{app}} . \end{aligned}$$

To show isotropy of the function $H^{2}$, we use the symmetry of the homogenization function $H$ (A.5), writing

A.12 $$\begin{aligned} \boldsymbol{R} \star H^{2}( \mathbb{C} ) &= \boldsymbol{R} \star \left (H( \mathbb{C} ) \otimes H( \mathbb{C} )\right ) \\ &= \left (H( \boldsymbol{R} \star \mathbb{C} ) \otimes H( \boldsymbol{R} \star \mathbb{C} )\right ) \\ &= H^{2}( \boldsymbol{R} \star \mathbb{C} ) \quad \text{for all} \quad \boldsymbol{R} \in S _{\mu _{L}}. \end{aligned}$$

In Eq. (A.6), it was shown that an $S _{\mu _{L}}$-invariant function has an $S _{\mu _{L}}$-invariant expectation. Consequently, the mean second moment $\langle{ {\mathbb{C}} ^{ \mathrm{app}} _{ L }\otimes \mathbb{C} ^{\mathrm{app}} _{ L }} \rangle _{L}$ is invariant with respect to the periodic symmetry group $S _{\mu _{L}}$.

The apparent fluctuation tensor (2.26) is given by

A.13 $$\begin{aligned} {\mathbb{Q}}^{8} _{ L }& =L^{3} \langle ( \mathbb{C} ^{\mathrm{app}} _{ L }- \langle \mathbb{C} ^{ \mathrm{app}} _{ L }\rangle _{ L }) \otimes ( \mathbb{C} ^{\mathrm{app}} _{ L }- \langle \mathbb{C} ^{\mathrm{app}} _{ L }\rangle _{ L })\rangle _{ L } \end{aligned}$$

A.14 $$\begin{aligned} & = L^{3} \langle \mathbb{C} ^{ \mathrm{app}} _{ L }\otimes \mathbb{C} ^{\mathrm{app}} _{ L } \rangle - \langle \mathbb{C} ^{ \mathrm{app}} _{ L }\rangle _{ L } \langle \mathbb{C} ^{\mathrm{app}} _{ L } \rangle _{ L }. \end{aligned}$$

As the expectations of the homogenization function $H$ and second-order homogenization function $H^{2}$ are invariant under the action of the group $S_{\mu _{L}}$, we find the invariance

A.15 $$\begin{aligned} \boldsymbol{R} \star {\mathbb{Q}}^{8} _{ L }&= \boldsymbol{R} \star \left (L^{3} \langle \mathbb{C} ^{\mathrm{app}} _{ L }\otimes \mathbb{C} ^{\mathrm{app}} _{ L }\rangle - \langle \mathbb{C} ^{ \mathrm{app}} _{ L }\rangle _{ L } \langle \mathbb{C} ^{\mathrm{app}} _{ L } \rangle _{ L }\right ) \end{aligned}$$

A.16 $$\begin{aligned} &= L^{3} \left ( \boldsymbol{R} \star \langle \mathbb{C} ^{ \mathrm{app}} _{ L }\otimes \mathbb{C} ^{\mathrm{app}} _{ L } \rangle - \left ( \boldsymbol{R} \star \langle \mathbb{C} ^{ \mathrm{app}} _{ L }\rangle _{ L }\right ) \otimes \left ( \boldsymbol{R} \star \langle \mathbb{C} ^{\mathrm{app}} _{ L } \rangle _{ L }\right ) \right ) \end{aligned}$$

A.17 $$\begin{aligned} &= L^{3} \left ( \langle \mathbb{C} ^{\mathrm{app}} _{ L } \otimes \mathbb{C} ^{\mathrm{app}} _{ L }\rangle - \langle \mathbb{C} ^{ \mathrm{app}} _{ L }\rangle _{ L }\otimes \langle \mathbb{C} ^{\mathrm{app}} _{ L } \rangle _{ L }\right ) \end{aligned}$$

A.18 $$\begin{aligned} &= {\mathbb{Q}}^{8} _{ L } \end{aligned}$$

for all ${ \boldsymbol{R} \in S _{\mu _{ L }}}$, i.e., the apparent fluctuation tensor ${\mathbb{Q}}^{8} _{ L }$ obeys the symmetry of the periodized ensemble. By considering a spherical domain $Y_{D}$ with Dirichlet boundary conditions (A.8), it follows that the fluctuation tensor ${\mathbb{Q}}^{8} $ has ensemble symmetry

A.19 $$ \boldsymbol{R} \star {\mathbb{Q}}^{8} = {\mathbb{Q}}^{8} \quad \text{for all} \quad \boldsymbol{R} \in S _{\mu}. $$
